# Microstructure, Length, and Connection of Limbic Tracts in Normal Human Brain Development

**DOI:** 10.3389/fnagi.2014.00228

**Published:** 2014-08-28

**Authors:** Qiaowen Yu, Yun Peng, Virendra Mishra, Austin Ouyang, Hang Li, Hong Zhang, Min Chen, Shuwei Liu, Hao Huang

**Affiliations:** ^1^Shandong Provincial Key Laboratory of Mental Disorders, Research Center for Sectional and Imaging Anatomy, Shandong University School of Medicine, Jinan, China; ^2^Advanced Imaging Research Center, University of Texas Southwestern Medical Center, Dallas, TX, USA; ^3^Department of Radiology, Beijing Children’s Hospital Affiliated to Capital Medical University, Beijing, China; ^4^Department of Mathematical Sciences, University of Texas at Dallas, Richardson, TX, USA; ^5^Department of Radiology, University of Texas Southwestern Medical Center, Dallas, TX, USA

**Keywords:** limbic tract, development, trajectory, length, microstructure, DTI, connectivity, free water elimination

## Abstract

The cingulum and fornix play an important role in memory, attention, spatial orientation, and feeling functions. Both microstructure and length of these limbic tracts can be affected by mental disorders such as Alzheimer’s disease, depression, autism, anxiety, and schizophrenia. To date, there has been little systematic characterization of their microstructure, length, and functional connectivity in normally developing brains. In this study, diffusion tensor imaging (DTI) and resting state functional MRI (rs-fMRI) data from 65 normally developing right-handed subjects from birth to young adulthood was acquired. After cingulate gyrus part of the cingulum (cgc), hippocampal part of the cingulum (cgh) and fornix (fx) were traced with DTI tractography, absolute and normalized tract lengths and DTI-derived metrics including fractional anisotropy, mean, axial, and radial diffusivity were measured for traced limbic tracts. Free water elimination (FWE) algorithm was adopted to improve accuracy of the measurements of DTI-derived metrics. The role of these limbic tracts in the functional network at birth and adulthood was explored. We found a logarithmic age-dependent trajectory for FWE-corrected DTI metric changes with fast increase of microstructural integrity from birth to 2 years old followed by a slow increase to 25 years old. Normalized tract length of cgc increases with age, while no significant relationship with age was found for normalized tract lengths of cgh and fx. Stronger microstructural integrity on the left side compared to that of the right side was found. With integrated DTI and rs-fMRI, the key connectional role of cgc and cgh in the default mode network was confirmed as early as birth. Systematic characterization of length and DTI metrics after FWE correction of limbic tracts offers insight into their morphological and microstructural developmental trajectories. These trajectories may serve as a normal reference for pediatric patients with mental disorders.

## Introduction

The limbic system is a group of interconnected cortical and subcortical structures dedicated to linking visceral states and emotion to cognition and behavior (Mesulam, 2000). The limbic system plays a key role in a variety of psychiatric and neuropsychological disorders including schizophrenia (e.g., Torrey and Peterson, 1974; Reynolds, 1983; White et al., 2008; Leech and Sharp, 2014), major depressive disorder (e.g., Monkul et al., 2007; DeRubeis et al., 2008; Duman and Voleti, 2012), autism (e.g., Amaral et al., 2008), Alzheimer’s disease (e.g., Braak and Braak, 1991), and obsessive-compulsive disorder (e.g., Fitzgerald et al., 2005; Abramowitz et al., 2009). As major white matter tracts underlying connectivity of the limbic system, cingulum bundle (including cingulum cingulate gyrus part or cgc and cingulum hippocampal part or cgh) and fornix (fx) could be affected in the pathological state. Moreover, structural changes of the limbic tracts could serve as an important biomarker for early detection of these disorders as previous studies suggest that disruption of limbic tracts precedes the clinical symptoms. For example, disruption of cgc was found in healthy subjects with high risk of major depressive disorder (e.g., Huang et al., 2011). Microstructural changes of fx, a major fiber bundle connecting to hippocampus, was found in subjects of mild cognitive impairment (MCI), a disorder that has been associated with risk for dementia (e.g., Oishi et al., 2011, 2012; Huang et al., 2012). Besides their important role in aging-related diseases such as dementia, disruption of limbic tracts was also found to be associated with psychiatric disorders of developing brain, such as schizophrenia (e.g., White et al., 2007) and autism (e.g., Weinstein et al., 2011). Quantitative characterization of these limbic tracts systematically during development thus offers a normal reference for mental disorders. Furthermore, understanding maturation of the limbic tracts may also provide insights on vulnerability of these tracts in dementia of aging brains. Although the uncinate fasciculus and anterior thalamic projections could also be considered as part of the limbic system white matter tracts (e.g., Catani et al., 2013), cgc/cgh and fx were focused in this study.

In the developing human brain, water molecules tend to diffuse more freely along the limbic tract, instead of perpendicular to it. This diffusion property can be measured non-invasively with diffusion MRI, a modality of MRI. Diffusion tensor imaging (DTI) (Basser et al., 1994) characterizes water diffusion properties with a tensor model. DTI-derived metrics are highly sensitive to white matter microstructural changes. Fractional anisotropy (FA) (Pierpaoli and Basser, 1996; Beaulieu, 2002), derived from DTI and quantifying the shape of diffusion tensor, has been widely used to characterize microstructural integrity. Radial diffusivity (RD) and axial diffusivity (AD), also derived from diffusion tensor, convey unique information related to myelination and axonal integrity, respectively (Song et al., 2002). Mean diffusivity (MD) is the linear combination of AD and RD. Limbic tracts can be non-invasively traced with tractography based on diffusion MRI (e.g., Conturo et al., 1999; Jones et al., 1999a; Mori et al., 1999; Basser et al., 2000; Catani et al., 2002).

With traced limbic tracts as binary masks for DTI-derived metric maps, the microstructural properties of the limbic tracts can be quantified. Characterization of structural development of limbic tracts has been included in recent studies on white matter development (e.g., Hermoye et al., 2006; Lebel and Beaulieu, 2011). With different developmental period focused in these studies, different curves for trajectories of limbic tract microstructural changes were reported. Thus, far systematic microstructural measurements of limbic tracts with age coverage from birth to 25 years old has not been found in the literature. Moreover, corrections of bias of DTI metric measurements caused by partial volume effects (PVE) have not been reported either. As limbic tracts are relatively thin bundles compared to other major tracts such as corpus callosum and pathways of cgh and fx are close to the ventricle, contamination of cerebrospinal fluid (CSF) on DTI metric measurements could be severe. Such contamination may not be consistent during development as more water content was found in the younger brains such as neonate brain (e.g., Neil et al., 1998; Mukherjee et al., 2001, 2008). Corrections need to be made for a more accurate estimate of developmental trajectories of limbic tract DTI metrics. To the best of our knowledge, morphology of limbic tracts has not been quantitatively characterized. As coherent fiber bundles, it is possible to measure geodesic lengths of limbic tracts based on tractography results.

The limbic tracts are also involved in functional connectivity. It has been reported that cgc and cgh play a vital role in adult brain default mode network (DMN) (van den Heuvel et al., 2008, 2009; Greicius et al., 2009; Uddin et al., 2009). Specifically, cgc connects medial prefrontal cortex (MPFC) and posterior cingulate cortex (PCC) and cgh connects PCC and medial temporal lobes (MTL). Functional connectivity in DMN is related to episodic memory, theory of mind, and the ability to “mentalize” (Fair et al., 2008). Appearance of functional connectivity in DMN has been reported as early in development as the neonate stage (Doria et al., 2010). But weak relationship between functional and structural connectivity within the DMN exists during childhood (Supekar et al., 2010). To understand the relationship between structure and function, it is important to confirm involvement of limbic tracts in connecting functional regions in the DMN as early as birth.

In this study, DTI from 65 normally developing right-handed subjects at cross-sectional ages from birth to young adulthood and resting state functional MRI (rs-fMRI) from 15 of the subjects were acquired. After cgc, cgh, and fx were traced with DTI tractography, absolute and normalized tract lengths and DTI-derived metrics including FA, MD, AD, and RD were measured for traced limbic tracts. Free water elimination (FWE) algorithm (Pasternak et al., 2009) was adopted to enhance accuracy of the measurements of DTI-derived metrics. The role of these limbic tracts in the functional network at birth and adulthood was explored. Systematic characterization of tract geodesic length and DTI metrics after FWE correction of limbic tracts offers insight into their morphological and microstructural developmental trajectories. These trajectories may serve as a normal reference for pediatric patients with mental disorders.

## Materials and Methods

### Human subjects

**Figure 1:**
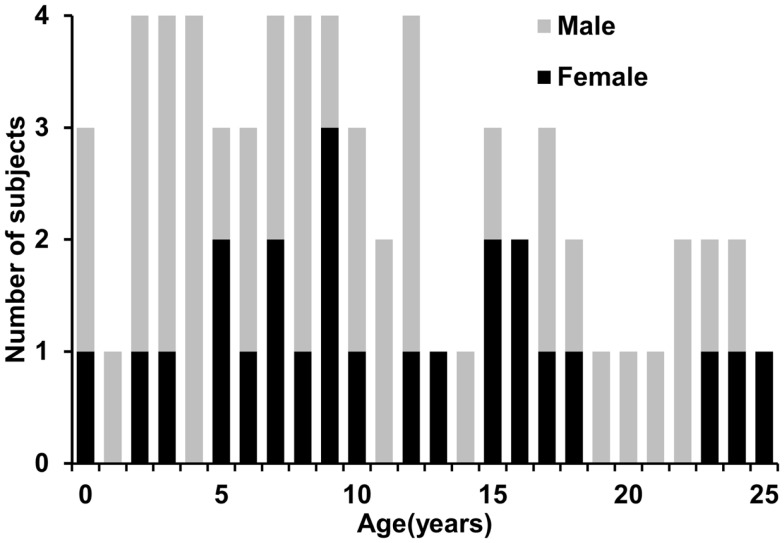
**Age and gender distribution of the 65 normal developing human subjects**.

Sixty-five healthy human subjects (41 M/24 F) with age range from 0 month (birth) to 25 years participated in this study. The age range covered the important developmental periods of early childhood, childhood, adolescence, and young adulthood. Figure 1 shows a histogram of the age and gender distribution of all subjects. Included neonates were part of the cohort for studying normal perinatal and prenatal brain development and were selected after rigorous screening procedures (Huang et al., 2014). Neonates were well fed before scanning. All included children, adolescents and young adults were healthy subjects free of current and past neurological or psychiatric disorders. Right-handed were reported for all children who showed clear handedness. For young children, besides earplugs and earphones, extra foam padding was applied to reduce the sound of the scanner while they were asleep. Due to difficulty of acquiring MRI from human subjects with such a comprehensive developmental period from 0 month to 25 years in one site, the presented data were acquired from the same type of MR scanner with common MRI protocol in two sites, Children’s Medical Center at Dallas and Children’s Hospital in Beijing. All subjects gave informed written consent approved by the Institutional Review Board of those two institutions.

### Data acquisition of DTI and fMRI

MRI of 3 neonates (2 M/1 F) at 40 weeks of gestation and 10 adolescents (12–17 years old; 4 M/6 F) was acquired from Children’s Medical Center at Dallas. MRI of all other subjects was acquired from Children’s Hospital in Beijing. A Philips 3 T Achieva MR scanner at both sites was used. A single-shot echo planar imaging (EPI) with SENSE (SENSitivity Encoding, reduction factor = 2.5) parallel imaging scheme was adopted for DTI acquisition. Eight-channel SENSE head coil and consoles installed with R2.6.3 software were used for both sites. The DTI imaging parameters were: TR (repetition time) = 6850 ms, TE (echo time) = 78 ms, in-plane field of view = 168 mm × 168 mm for neonates and 224 mm × 224 mm for all others, in-plane imaging resolution = 1.5 mm × 1.5 mm for neonates and 2 mm × 2 mm for all others, slice thickness = 1.5 mm for neonate to 2 mm for all others, slice number = 60–65 depending on the height of the subject brains, 30 independent diffusion-weighted directions (Jones et al., 1999b) uniformly distributed in space, *b*-value = 1000 s/mm^2^, and repetition = 2. The axial image dimension was 256 × 256 after reconstruction. The total acquisition time of DTI was 11 min. With 30 diffusion weighted image (DWI) volumes and 2 repetitions, we accepted only those scanned datasets with less than 5 DWI volumes affected by motion more commonly seen in scan of neonates and young children. The affected volumes were replaced by the good volumes of another DTI repetition during postprocessing. rs-fMRI was also acquired from 15 out of 65 subjects, including 3 neonates and 3 young adults. A T2* weighted gradient echo EPI sequence was used. A total of 210 T2* weighted whole brain volumes were acquired with the following parameters: TR = 1500 ms for neonates and 2000 ms for young adults, TE = 27 ms for neonates and 30 ms for young adults, flip angle = 80°, in-plane imaging resolution = 2.4 mm × 2.4 mm for neonates and 3.4 mm × 3.4 mm for young adults, and slice thickness = 3mm with no gap for neonates and 4 mm with no gap for young adults; slice number = 30 for neonates and 37 for young adults. Scan time of rs-fMRI was 5–6 min. Rs-fMRI and DTI images were acquired in the same session.

### Tensor fitting and DTI-based tractography

Diffusion weighted images for each subject were corrected for motion and eddy current by registering all DWIs to the b0 image using a 12-parameter (affine) linear registration with automated image registration (AIR) algorithm (Woods et al., 1998). After the registration, six independent elements of the 3 × 3 diffusion tensor (Basser et al., 1994) were determined by multivariate least-square fitting of DWIs. The tensor was diagonalized to obtain three eigenvalues ($\lambda_{1-3}$) and eigenvectors $\left(v_{1-3}\right)$. FA, MD, AD, and RD, derived from DTI and used to quantify white matter microstructure, were obtained for all the subjects using the equations of eigenvalues described in details previously (e.g., Mishra et al., 2013).

Fiber assignment of continuous tractography (FACT) (Mori et al., 1999) was used to trace limbic tracts for all subjects. The limbic tracts include cgc, cgh, and fx. FA threshold of 0.2 and a principal eigenvector turning angle threshold of 50 were used for FACT tractography. cgc and cgh were traced following the fiber tracking protocol (Wakana et al., 2007) with a multiple-ROI approach (Huang et al., 2004). The ROI placement protocol for tracing fx was as follows. The first ROI was drawn to include main body of fx identifiable on an axial slice of DTI color-encoded map. The second ROI was placed on the coronal slice where hippocampus was identifiable using “AND” operation. All other fibers were removed carefully to retain only the fx. The above-mentioned tensor fitting and fiber tracking was conducted by using DTIStudio (Jiang et al., 2006).

### Measurements of lengths and DTI metrics of limbic tracts

After tractography of cgc, cgh, and fx, the lengths and DTI metrics of each tract were measured for all subjects and plotted against age. These limbic tracts were visualized in 3D with Amira software (FEI, Berlington, MA, USA).

#### Measurement of tract length

The absolute and normalized limbic tract length were measured based on the tractography results. Specifically, geodesic length of each fiber in cgc, cgh, and fx was measured. A histogram was established for each limbic tract to show the distribution of fiber length. Note that a tract contains fibers with different lengths. In order to reduce the bias caused by a large number of short fibers, we defined the tract length by averaging 10% longest fibers of each tract. As shorter fibers could only cover part of the tract, reconstruction of the top 10% longest fibers showed that morphology of these fibers was in good agreement with that of the entire tract of all fibers. Tract length was further normalized by the size of the brain represented by the length between most anterior to most posterior edge to quantify relative tract length change during development.

#### Measurements of uncorrected and corrected DTI-based metrics

The binary masks of the individually traced limbic system tracts were used to compute the tract-level FA, MD, AD, and RD without correction of PVE of free water, defined as uncorrected DTI metrics. Young brains contain a large amount of free water. The uncorrected DTI metrics reflect the weighted average of all compartments including free water. In order to minimize the influence of CSF, FWE (Pasternak et al., 2009) was applied to correct the DTI metrics of cgc, cgh, and fx by using the following model: $S=S_{0}\left(f_{\mathrm{iso}}e^{-bD_{\mathrm{iso}}}+\left(1-f_{\mathrm{iso}}\right)e^{-b\bar{D}}\right),$ where *S* is the measured signal in the voxel, *f*_iso_ is the volume fraction of the isotropic compartment, *b* is the applied *b*-value, *S*_0_ is the signal with no diffusion weighting, *D*_iso_ is the diffusion coefficient of the isotropic compartment, and $\bar{\bar{D}}$ is the corrected tensor representing the fiber bundle in the voxel. This is a two-compartment model with one compartment representing the fiber bundle and the other compartment representing the isotropic free water compartment in the voxel. We estimated the six elements of $\bar{\bar{D}}$ and *f*_iso_ by solving the non-linear equation in the model. The initial values of $\bar{\bar{D}}$ for iteration are those from single tensor model without isotropic component. The diffusivity of the isotropic compartment *D*_iso_ was 3 × 10^−3^ mm^2^/s (Pasternak et al., 2009). Note that with measurements from 30 diffusion gradients, an over-determined system was used to fit the six independent elements of tensor $\bar{\bar{D}}$ and *f*_iso_.

### Rs-fMRI data processing

The DMNs of three neonates and three young adults were identified with independent component analysis (ICA) of the rs-fMRI data. ICA was conducted with MELODIC tool (Multivariate Exploratory Linear Decomposition into Independent Components, a part of FSL)^1^ . Pre-processing procedures included removing the first 10 volumes for stabilization of the magnetic field, motion correction, spatial smoothing with a Gaussian kernel of FWHM 5 mm, intensity normalization, and high-pass temporal filtering. Volumes with translational movement greater than 5 mm were removed from the dataset. The DMN cluster components in the fMRI space were transformed into the DTI space by registering the resampled fMRI image to b0 image in the DTI space using SPM8 (Statistical Parametric Mapping 8)^2^ . Amira software (FEI, Berlington, MA) was used for reconstructing the DMN clusters and limbic tracts connecting them.

### Curve fitting and statistical analysis

The following equation was used for fitting a model between measurement *y* (including uncorrected/corrected DTI metrics and absolute/normalized tract length) and age *t, y* = *f*(*t*) + *c*·sex + ε, where *f*(*t*) was linear, logarithmic, or polynomial function of *t* and ε was an error term. *F*-test was used to find the best fitting curve. We first tested if the measurement *y* was age dependent, if so, we further tested which of the following curves, linear, logarithmic, or polynomial curves, fitted the data best.

With age and gender as covariates, generalized linear model (GLM) was used to test (1) if FWE correction significantly changed DTI metric measurements and (2) if lateralization (i.e., measurement in left or right hemisphere) was a significant factor for all measurements. The null hypothesis for FWE correction is that there is no difference of DTI metric measurement before and after the correction. The null hypothesis for lateralization is that there is no difference of length or DTI metric measurement of corresponding limbic tracts in the left and right hemisphere. The Bonferroni correction was used to control the spurious positives when rejecting the null hypotheses.

## Results

### 3D reconstructed limbic tracts at different developmental stages

**Figure 2:**
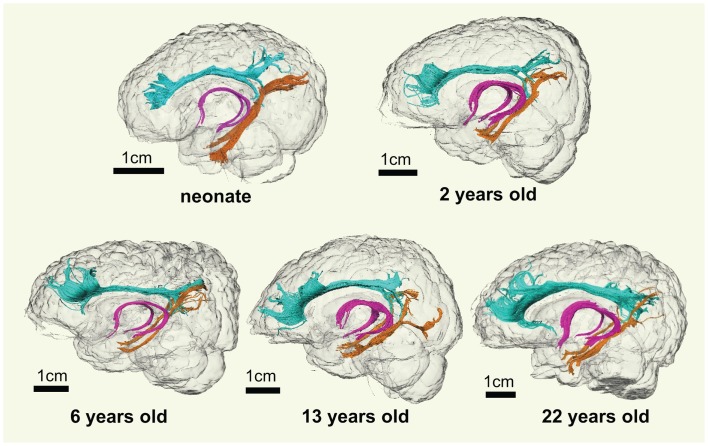
**3D visualization of the traced limbic tracts of representative subjects at following developing stages, at birth (0 month), early childhood (2 years old), childhood (6 years old), adolescence (13 years old), and young adulthood (22 years old)**. Cingulate gyrus part of cingulum (cgc), cingulum hippocampal part (cgh), and fornix (fx) were painted with cyan, orange, and pink color, respectively.

As shown in Figure 2, overall consistent 3D reconstructed limbic tracts, cgc, cgh, and fx, can be observed at five time points throughout the developmental period from 0 month to 25 years. cgc appears to be more extended to prefrontal regions and has more branches during development, while morphology of cgh and fx remains relatively stable.

### Development of microstructures of limbic tracts

**Figure 3:**
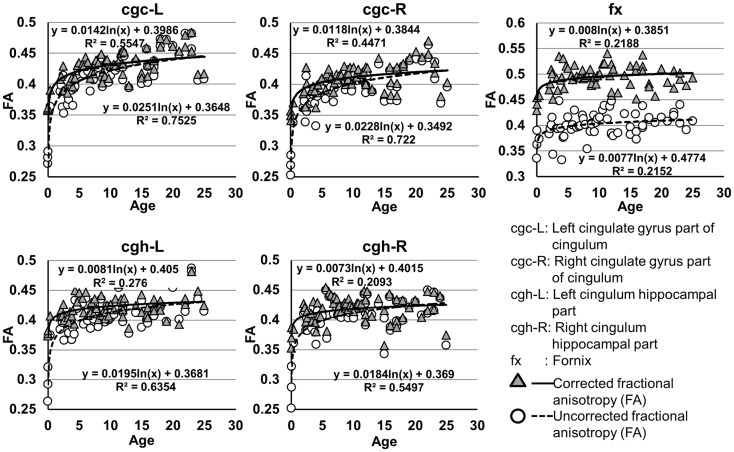
**Age-dependent changes of uncorrected and corrected fractional anisotropy (FA) measurements of limbic tracts**. *R*^2^ and fitting equations are listed in each panel.

**Figure 4:**
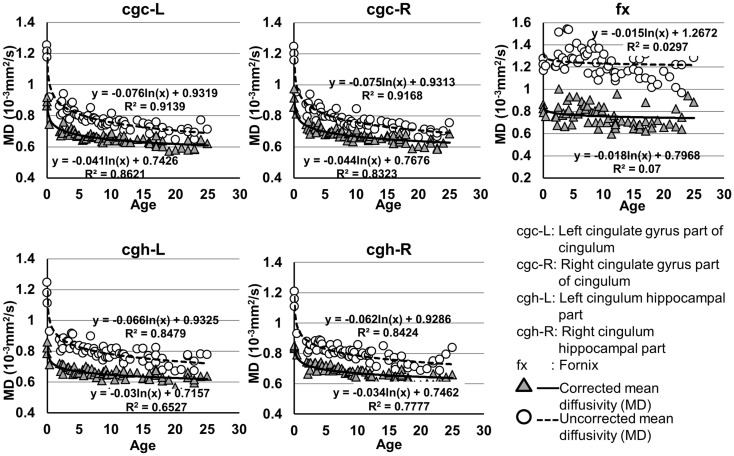
**Age-dependent changes of uncorrected and corrected mean diffusivity (MD) measurements of limbic tracts**. *R*^2^ and fitting equations are listed in each panel.

**Figure 5:**
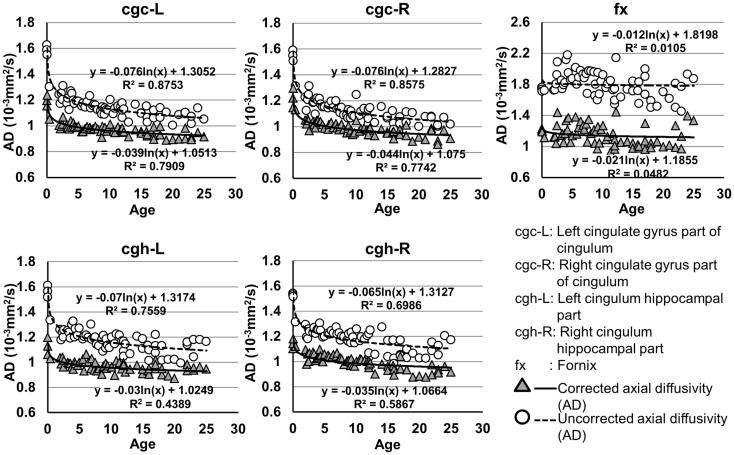
**Age-dependent changes of uncorrected and corrected axial diffusivity (AD) measurements of limbic tracts**. *R*^2^ and fitting equations are listed in each panel.

**Figure 6:**
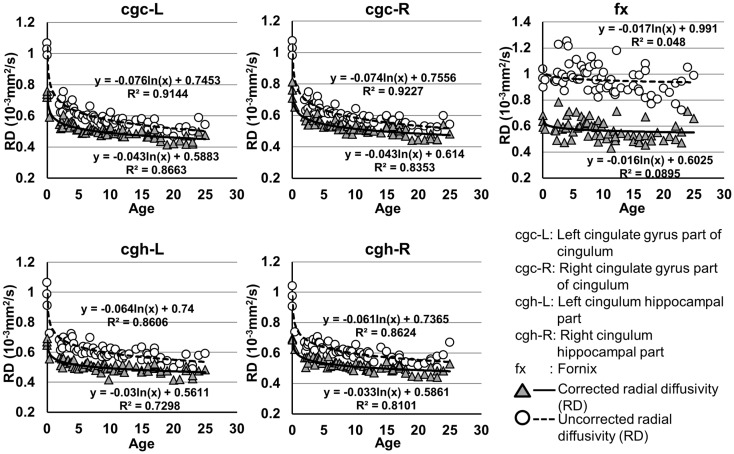
**Age-dependent changes of uncorrected and corrected radial diffusivity (RD) measurements of limbic tracts**. *R*^2^ and fitting equations are listed in each panel.

The plots of uncorrected and corrected DTI metrics, namely, FA, MD, AD, and RD, of all limbic tracts at different ages are shown in Figures 3–6, respectively. As shown in Tables 1–4, significant age dependence was found in almost all DTI metrics of all limbic tracts, except measurements of MD, AD, and RD of fx. Among logarithmic, linear, and polynomial fitting between uncorrected/corrected DTI metrics and age, logarithmic model fits best to most of DTI metric measurements of the limbic tract, also shown in Tables 1–4. For rest of the fittings, there is no statistical difference between linear and logarithmic fitting. No significant difference between linear and logarithmic fitting is especially apparent for corrected FA measurements of all limbic tracts (Table 1). Lowest *R*^2^ was obtained with the polynomial fitting.

**Table 1:** **Relationship between fractional anisotropy (FA) of limbic tracts and age**.

Limbic tracts	Age dependence	*F*-test between linear and logarithmic model		Best-fitting curve
		*F* value	*p* value	
cgc-L (UC)	***	2.11	**	Logarithmic
cgc-R (UC)	***	2.23	**	Logarithmic
cgh-L (UC)	***	1.87	*	Logarithmic
cgh-R (UC)	***	1.9	*	Logarithmic
fx (UC)	**	1.1	NS	Logarithmic/linear
cgc-L (C)	***	1.21	NS	Logarithmic/linear
cgc-R (C)	***	1.24	NS	Logarithmic/linear
cgh-L (C)	***	1.1	NS	Logarithmic/linear
cgh-R (C)	***	1.21	NS	Logarithmic/linear
fx (C)	**	1.24	NS	Logarithmic/linear

Statistical significance *p* < 0.05, *p* < 0.01, and *p* < 0.0001 are indicated by bold *, **, and ***, respectively. cgc, right cingulate gyrus part of cingulum; cgh, hippocampal part of cingulum; fx, fornix; UC, uncorrected; C, corrected; L, left; R, right; NS, not significant.

**Table 2:** **Relationship between mean diffusivity (MD) of limbic tracts and age**.

Limbic tracts	Age dependence	*F-*test between linear and logarithmic model		Best-fitting curve
		*F* value	*p* value	
cgc-L (UC)	***	6.12	***	Logarithmic
cgc-R (UC)	***	6.16	***	Logarithmic
cgh-L (UC)	***	3.75	***	Logarithmic
cgh-R (UC)	***	3.49	***	Logarithmic
fx (UC)	NS	–	–	–
cgc-L (C)	***	3.5	***	Logarithmic
cgc-R (C)	***	2.83	***	Logarithmic
cgh-L (C)	***	1.75	*	Logarithmic
cgh-R (C)	***	1.67	**	Logarithmic
fx (C)	NS	–	–	–

Statistical significance *p* < 0.05, *p* < 0.01, and *p* < 0.0001 are indicated by bold *, **, and ***, respectively. cgc, right cingulate gyrus part of cingulum; cgh, hippocampal part of cingulum; fx, fornix; UC, uncorrected; C, corrected; L, left; R, right; NS, not significant.

**Table 3:** **Relationship between axial diffusivity (AD) of limbic tracts and age**.

Limbic tracts	Age dependence	*F-*test between linear and logarithmic model		Best-fitting curve
		*F* value	*p* value	
cgc-L (UC)	***	4.37	***	Logarithmic
cgc-R (UC)	***	3.57	***	Logarithmic
cgh-L (UC)	***	2.34	**	Logarithmic
cgh-R (UC)	***	1.7	NS	Logarithmic/linear
fx (UC)	NS	–	–	–
cgc-L (C)	***	2.6	**	Logarithmic
cgc-R (C)	***	2.15	**	Logarithmic
cgh-L (C)	***	1.28	NS	Logarithmic
cgh-R (C)	***	0.78	NS	Logarithmic/linear
fx (C)	NS	–	–	–

Statistical significance *p* < 0.05, *p* < 0.01, and *p* < 0.0001 are indicated by bold *, **, and ***, respectively. cgc, right cingulate gyrus part of cingulum; cgh, hippocampal part of cingulum; fx, fornix; UC, uncorrected; C, corrected; L, left; R, right; NS, not significant.

**Table 4:** **Relationship between radial diffusivity (RD) of limbic tracts and age**.

Limbic tracts	Age dependence	*F*-test between linear and logarithmic model		Best-fitting curve
		*F* value	*p* value	
cgc-L (UC)	***	6.16	***	Logarithmic
cgc-R (UC)	***	6.81	***	Logarithmic
cgh-L (UC)	***	4.25	***	Logarithmic
cgh-R (UC)	***	4.41	***	Logarithmic
fx (UC)	NS	–	–	–
cgc-L (C)	***	3.55	***	Logarithmic
cgc-R (C)	***	2.93	***	Logarithmic
cgh-L (C)	***	2.14	**	Logarithmic
cgh-R (C)	***	2.49	**	Logarithmic
fx (C)	*	0.99	NS	Logarithmic/linear

Statistical significance *p* < 0.05, *p* < 0.01, and *p* < 0.0001 are indicated by bold *, **, and ***, respectively. cgc, right cingulate gyrus part of cingulum; cgh, hippocampal part of cingulum; fx, fornix; UC, uncorrected; C, corrected; L, left; R, right; NS, not significant.

As shown in Table 5, DTI metrics of all limbic tracts were significantly altered after FWE correction. FA increases due to FWE correction for fx is apparent in Figure 3, while uncorrected and corrected FA of cgc-L/R and cgh-L/R overlap with no visible changes. Decrease of MD, AD, and RD of all limbic tracts can be clearly observed in Figures 3–6. Statistical results in Table 5 indicate that there are statistically significant changes for all DTI metrics of all limbic tracts with FWE correction.

**Table 5:** **Comparisons of DTI metrics of limbic tract before and after FWE correction with age and gender as covariates**.

Limbic tracts	FA		AD		RD		MD	
	*t* value	*p* value	*t* value	*p* value	*t* value	*p* value	*t* value	*p* value
cgc-L	88.28	***	341.8	***	287	***	324	***
cgc-R	73.44	***	264.9	***	281	***	298.1	***
cgh-L	40.82	***	207.1	***	208.3	***	229.2	***
cgh-R	28.63	***	191.3	***	232.4	***	254.5	***
fx	214.5	***	214.3	***	191.8	***	206.5	***

Statistical significance after Bonferroni correction is indicated by bold. cgc, right cingulate gyrus part of cingulum; cgh, hippocampal part of cingulum; fx, fornix; L, left; R, right; ***p < 0.001.

### Development of lengths of limbic tracts

**Figure 7:**
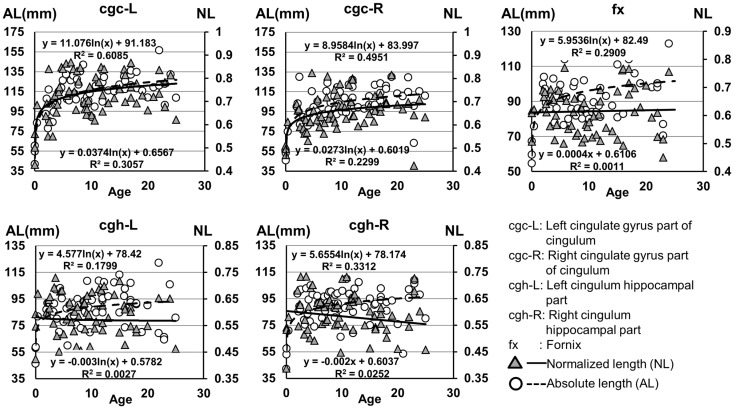
**Age-dependent changes of absolute and normalized fiber length of limbic tracts**. *R*^2^ and fitting equations are listed in each panel.

The plots of absolute and normalized lengths of all limbic tracts at different ages are shown in Figure 7. As shown in Table 6, significant age dependence was found in absolute lengths of all limbic tracts and normalized lengths of cgc-L/R. Among logarithmic, linear, and polynomial fitting between measured length and age, polynomial model was first rejected due to lowest *R*^2^. After *F*-test, significant difference between logarithmic and linear model was found only for absolute length of cgc-L (Table 6). Logarithmic curves were fitted with highest *R*^2^ for absolute and normalized lengths of all limbic tracts, as shown in dashed and solid curves in Figure 7, respectively. Figure 7 and Table 6 suggest that increases of absolute tract lengths of cgh-L, cgh-R, and fx results from overall brain size increases during development, while increases of absolute tract length of cgc-L and cgc-R are due to both overall brain size increases and relative increases of lengths of these two tracts in the brain.

**Table 6:** **Relationship between limbic tract length (absolute and normalized) and age**.

Tract length	Age dependence	*F*-test between linear and logarithmic		Best-fitting curve
		*F* value	*p* value	
cgc-L (absolute)	***	1.79	*	Logarithmic
cgc-R (absolute)	***	1.46	NS	Logarithmic
cgh-L (absolute)	***	1.19	NS	Logarithmic/linear
cgh-R (absolute)	***	1.39	NS	Logarithmic/linear
fx (absolute)	***	1.17	NS	Logarithmic/linear
cgc-L (normalized)	***	1.32	NS	Logarithmic
cgc-R (normalized)	***	1.2	NS	Logarithmic
cgh-L (normalized)	NS	–	–	–
cgh-R (normalized)	NS	–	–	–
fx (normalized)	NS	–	–	–

Statistical significance is indicated by bold. cgc, right cingulate gyrus part of cingulum; cgh, hippocampal part of cingulum; fx, fornix; L, left; R, right; *p < 0.05; ***p < 0.001; NS, not significant.

### Lateralization

Higher FA and lower AD, RD, and MD in the left cgc/cgh can be observed in Figures 3–6. In addition, longer normalized tract length of cgc on the left side compared to that on the right side can be observed in Figure 7. Statistical comparisons of normalized tract length and corrected DTI metrics on the left and right side are listed in Table 7. Significant differences were found in all DTI metrics between the left and right cgc and between the left and right cgh (Table 7). Statistically significant difference of normalized tract length was found between cgc-L and cgc-R, but not between cgh-L and cgh-R (Table 7).

**Table 7:** **Comparisons of normalized tract length and corrected DTI metrics between the left and right cingulate gyrus part of cingulum (cgc) and between the left and right hippocampal part of cingulum (cgh), with age and gender as covariates**.

Corrected DTI metrics	Fractional anisotropy		Axial diffusivity		Radial diffusivity		Mean diffusivity	
	*t* value	*p* value	*t* value	*p* value	*t* value	*p* value	*t* value	*p* value
cgc-L vs cgc-R	68.8	***	150.8	***	253.4	***	238.5	***
cgh-L vs cgh-R	14.2	***	50.94	***	149.2	***	115.2	***

**Normalized tract length**	***t* value**				***p* value**

cgc-L vs cgc-R	30.1				***
cgh-L vs cgh-R	0.75				NS

Statistical significance *p* < 0.001 after Bonferroni correction is indicated by bold ***. L, left; R, right; NS, not significant.

### Structural connectivity of DMN regions through limbic tracts for neonate and adult brain

**Figure 8:**
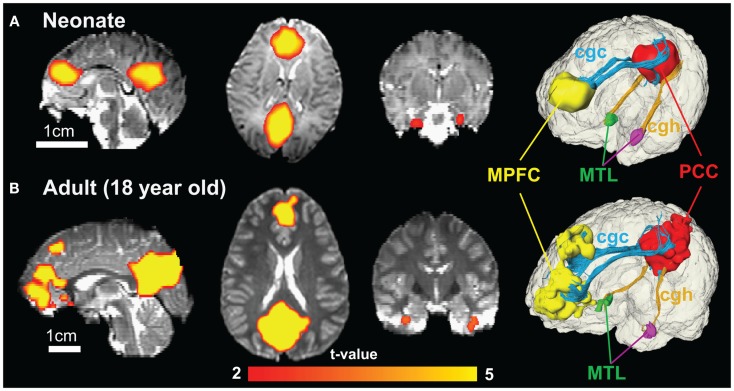
**Functional connectivity maps of DMN in sagittal, axial, and coronal slices from the left to right show similar connectivity pattern in neonate (A) and adult (B) brain**. The color of connected brain clusters encodes *t* values. 3D visualization on the most right panels reveals clearly that cingulate gyrus part of cingulum (cgc) connects MPFC and PCC and cingulum hippocampal part (cgh) connects PCC and MTL for both neonate and adult brain. The 3D reconstruction of clusters at MPFC, PCC, and MTL was directly extracted from rs-fMRI analysis.

Figure 8 shows the functional connectivity maps in the DMN and structural limbic tracts connecting the DMN regions for a neonate and an adult brain. Consistency of DMN regions (MPFC, PCC, and MTL) and limbic tracts connecting these regions is clear between the neonate (Figure 8A) and adult (Figure 8B) brain. Specifically, cgc connects MPFC and PCC; cgh connects PCC and MTL for both neonate and adult brain.

## Discussion

With DTI from 65 subjects of cross-sectional age from 0 month to 25 years and rs-fMRI from 15 of them, we have quantitatively characterized development of microstructure, length, and connection of limbic tracts that play a key role in emotion, memories, and behavior. Limbic tracts were traced with DTI tractography. As limbic tracts are relatively thin and discrete compared to other major white matter tracts such as corpus callosum, the contamination of CSF to DTI metric measurements cannot be ignored. Correction of DTI metric measurement with FWE was conducted to achieve more accurate measurements of limbic tract microstructure. Together with the tract length measurement, microstructure and length of limbic tracts from birth to adulthood were quantitatively characterized. In addition, consistent role of limbic tracts connecting major DMN regions at birth and adulthood has been confirmed and demonstrated. Comparisons between DTI metrics and lengths of the left and right limbic tracts suggest significant lateralization with longer and better myelinated cgc on the left side compared to that on the right side.

Despite that the overall shape of limbic tracts is relatively consistent throughout the developmental period from birth to young adulthood (Figure 2), Figures 3–6 demonstrate rapid increase of FA and decrease of AD, RD, and MD within the first 2 years and relatively slow changes of these DTI metrics after 2 years until a plateau is reached during adolescence. Age-dependent increase of FA could be caused by larger decrease of RD compared to that of AD as FA is roughly the ratio between AD and RD. Rapid FA changes of cingulum within first 2 years could be caused by myelination, the majority of which begins within the first 2 years of life (Yakovlev and Andre-Roch, 1967). The asynchronous myelination of white matter tracts has been reported in neuropathology (Kinney et al., 1988) and MRI study (e.g., Deoni et al., 2011). Memory, emotion, and motivation functions are related to limbic tracts and important for survival. It is vital for limbic tracts to become well myelined earlier than other tracts, especially those projected from frontal and temporal lobes (Baumann and Pham-Dinh, 2001).

Including cross-sectional ages from birth to 25 years with a consistent group of dataset could offer more complete insight into structural maturation of limbic tracts. After test of three models including linear, logarithmic, and polynomial, Tables 1–4 show that logarithmic model is overall best fitted to most limbic tract microstructural trajectories. Microstructural measurements of developing limbic tracts were found in previous literature (e.g., Hermoye et al., 2006; Grieve et al., 2011; Lebel and Beaulieu, 2011), which includes less comprehensive developmental periods. Depending on the early or late developing time window focused in these previous studies, different curves for developmental trajectories of limbic tracts were reported. For example, a non-linear trend of DTI metrics of white matter tracts was reported in a longitudinal DTI study with age range from 5 to 32 years (Lebel and Beaulieu, 2011). In that study, a large number of subjects were enrolled with 2 scans for most of the subjects, but early childhood period from 0 to 5 years was absent. During the early stage of development from birth to 54 months, non-linear trends could also be observed from measurements of FA and averaged apparent diffusion coefficient of cingulum (Hermoye et al., 2006), showing rapid DTI metric change in the first 3–6 months. A linear model was reported to be more suitable for very early development of limbic tracts of infants with shorter age range from 3.9 to 18.4 weeks (Dubois et al., 2008). In another study with later stage of limbic tract development from childhood to adulthood, polynomial curve was reported (Grieve et al., 2011). These previous findings are generally in agreement with the results presented in this study, since segmented developmental trajectory of DTI metrics at different developmental time window from birth to childhood could be quite different. For example, it can be observed from Figures 3–6 that limbic tract metric changes before 2 years demonstrate clear linear behavior.

cgc-L/R, cgh-L/R, and fx are relatively thin and discrete white matter tracts. Contamination of brain CSF to DTI metric measurements of the limbic tracts could be severe. We adopted FWE (Pasternak et al., 2009) to achieve a more accurate characterization of developmental trajectories of microstructural measures. It is clear that FA is increased and AD, RD, and MD are decreased after FWE correction, causing clear shift of DTI metric trajectories in Figures 3–6. Results in Table 5 confirmed statistical significance of the shift after FWE correction. The amount of correction of DTI metric measurement is more dramatic for fx compared to that of cgc or cgh, as fibers of fx course closely around the ventricle and metric measurements of fx could be affected by severe CSF contamination. FA trajectories of cgc-L/R and cgh-L/R (Figure 3) are less affected by FWE correction than those of MD, AD, and RD (Figures 3–6) as FA can be considered approximately as a ratio of AD and RD and shift of AD and shift of RD offset each other. The shifts between uncorrected and corrected curves in Figures 3–6 were heterogeneous. Specifically, DTI metric measurements of cgc-L/R and cgh-L/R were more severely affected by free water contamination in early childhood (Figures 3–6). Nevertheless, these shifts did not change the fitting models for most limbic tracts (Tables 1–4). Only for corrected FA of cgc-L/R and cgh-L/R, developmental trajectories of them could also be fitted with linear model as well as logarithmic model (Table 1) after FWE correction. To the best of our knowledge, no FWE correction has been reported for previous DTI metric measurements of developing limbic tracts. It is possible that FWE correction could help restore the real underlying microstructural maturation process of limbic tracts. Nevertheless, further cross-validation from a larger sample dataset is still needed to ensure accuracy of DTI metric measures after FWE correction.

Lengths of all limbic tracts increase during development, as shown in Figure 7 and Table 6. Specifically, the length increases of cgh-L/R and fx follow the overall brain size development and no significant age dependence has been found after these lengths were normalized by the brain size. Significant age dependence has been found only for normalized lengths of cgc-L/R, indicating that there are extra length increases of cgc-L/R besides following growth of entire brain. Although the overall shape of cgc is relatively stable throughout development, extra cgc growth can be observed in its anterior part close to prefrontal cortex (Figure 2). Relative increase of cgc length is probably related to its growth in the prefrontal region. Functions of prefrontal areas are involved in planning, decision making, and moderating social behavior that develop during late childhood and adolescence (e.g., Gogtay et al., 2004). Connection of anterior cgc to late-developed prefrontal cortex may explain extra length growth of cgc from birth to young adulthood, but no extra length growth of cgh or fx.

Significant lateralization has been found for all DTI metrics of cgc-L/R and cgh-L/R with age and gender as covariates, as shown in Table 7. Higher FA and lower AD, RD, and MD of cgc and cgh can be observed on the left side than right side (Figures 3–6). This lateralization was associated with higher microstructural integrity on the left side of limbic tracts. Lateralization of DTI metrics of cgc and cgh may be related to unique functions of the left side of human brain such as language (van Veen et al., 2001). Exclusive right-handedness of the recruited subjects may also play a role. These findings are consistent to previous DTI metric measurements of cingulum (Gong et al., 2005; Verhoeven et al., 2010). Strengthened left limbic tracts were also demonstrated by longer length of cgc-L compared to that of cgc-R (Table 7). However, no significant length difference could be found for the left and right cgh.

In functional connectivity, brain regions where spontaneous blood oxygen level-dependent (BOLD) signal oscillations are temporally correlated can be either directly connected by white matter tracts or connected through a relay. The adult brain regions of PCC, MPFC, and MTL in DMN are connected by limbic tracts (Greicius et al., 2009). Figure 8 clearly demonstrates consistent connectivity roles of limbic tracts in connecting DMN regions for both the neonate and adult brain. Immature DMN functional connectivity has been reported with rs-fMRI of neonate brains recently (Fransson et al., 2007, 2009; Doria et al., 2010; Smyser et al., 2010). Unlike other major white matter tracts such as arcuate fasciculus, limbic tracts are well formed at birth, as shown in Figure 2. However, the role of limbic tracts in connecting DMN regions as early as birth time has not been confirmed previously. With myelination of cgc and cgh during development indicated by Figures 3–6, it can be speculated that enhanced myelination of cgc and cgh provide structural basis for maturation of DMN during development.

It is difficult to acquire all DTI data from birth to young adulthood in one site. We combined datasets acquired in two sites, but with the same imaging protocol and same Philips 3 T Achieva scanners. Rigorous quality control is conducted routinely for both scanners. We performed quantitative analysis based on DTI tractography and metric measurements to evaluate the effects of these two scanners in our previous study (Huang et al., 2014), and it was concluded that the scanner effects are negligible. Multiple factors could contribute to variations of MRI data, including variability caused by thermal variations of the same scanner itself, physiological variability of the participant and differences of two scanners. In an earlier experiment, DTI was acquired from the same young adult human volunteer scanned at two Philips 3 T Achieva scanners. FA measurement differences caused by scanner difference were tested to be within the range of variability of scanning the same subject twice with one scanner (Saxena et al., 2012). These previous experiments suggest that scanner difference will not cause significant differences of reported results. It also should be noted that this study is not a real longitudinal one as the subjects at each cross-sectional age were not the same. It will be very difficult to follow a same cohort of subjects from birth to 25 years old. However, multiple scans from same subjects during their development could be helpful to characterize real longitudinal development of limbic tracts.

In conclusion, with DTI and rs-fMRI from developing brains at cross-sectional ages from 0 month to 25 years, we have quantitatively characterized development of microstructure, length, and connection of limbic tracts that play a key role in emotion, memories, and behavior. Logarithmic developmental trajectories were found for most age-dependent changes of limbic tract microstructures and lengths from birth to young adulthood. Correction of CSF contamination has significantly increased FA and reduced MD, AD, and RD of developing limbic tracts, but has not changed the age-dependent trajectories of these metrics. Stronger microstructural integrity and longer tract lengths were found for limbic tracts on the left side compared to those on the right side. Consistent role of limbic tracts connecting major DMN regions at birth and adulthood has been confirmed and demonstrated.

## Conflict of Interest Statement

The authors declare that the research was conducted in the absence of any commercial or financial relationships that could be construed as a potential conflict of interest.

## Supplementary Material

The Supplementary Material for this article can be found online at http://www.frontiersin.org/Journal/10.3389/fnagi.2014.00228/abstract

## Footnotes

^1^www.fmrib.ox.ac.uk/fsl/melodic2/index.html

^2^http://www.fil.ion.ucl.ac.uk/spm/
