# Supplementary material for: Microstructure, Length, and Connection of Limbic Tracts in Normal Human Brain Development
Source: Front Aging Neurosci. 2014 Aug 28;6:228. doi: 10.3389/fnagi.2014.00228 (PMC4147394; doi:10.3389/fnagi.2014.00228)
Supplement: Supplementary file 1 [file Data_Sheet1.DOCX]

Supplemental Table 1: Fractional anisotropy (FA) of each subject before and after free water elimination represented by UC (uncorrected) and C (corrected), respectively. The FA values for left (L) and right (R) cingulate gyrus part of cingulum (cgc), left and right cingulum hippocampal part (cgh) and fornix (fx) are shown. This table shows the quantitative data of Fig. 3.

| **Sub ID** | **Age (Years)** | **cgc-L** | | **cgc-R** | | **cgh-L** | | **cgh-R** | | **fx** | |
| --- | --- | --- | --- | --- | --- | --- | --- | --- | --- | --- | --- |
|  |  | **FA (UC)** | **FA (C)** | **FA (UC)** | **FA (C)** | **FA (UC)** | **FA (C)** | **FA (UC)** | **FA (C)** | **FA (UC)** | **FA (C)** |
| Sub#1 | 0.02 | 0.27141 | 0.35741 | 0.25274 | 0.35652 | 0.26363 | 0.37322 | 0.25213 | 0.35272 | 0.33551 | 0.42880 |
| Sub#2 | 0.03 | 0.28123 | 0.35950 | 0.26887 | 0.34902 | 0.29226 | 0.37768 | 0.28686 | 0.36985 | 0.36268 | 0.44154 |
| Sub#3 | 0.05 | 0.29104 | 0.36159 | 0.28501 | 0.34151 | 0.32090 | 0.38213 | 0.32159 | 0.38698 | 0.38985 | 0.45428 |
| Sub#4 | 0.42 | 0.36346 | 0.39010 | 0.33783 | 0.36996 | 0.37479 | 0.40692 | 0.36092 | 0.38434 | 0.37330 | 0.45798 |
| Sub#5 | 1.99 | 0.38787 | 0.40779 | 0.36161 | 0.38461 | 0.38431 | 0.40561 | 0.41031 | 0.42726 | 0.38324 | 0.46956 |
| Sub#6 | 2.17 | 0.39811 | 0.41287 | 0.37287 | 0.38775 | 0.40481 | 0.41752 | 0.41366 | 0.42089 | 0.39317 | 0.48113 |
| Sub#7 | 2.35 | 0.36272 | 0.38836 | 0.33866 | 0.36322 | 0.39553 | 0.40725 | 0.38253 | 0.39659 | 0.40083 | 0.50881 |
| Sub#8 | 2.50 | 0.41414 | 0.42911 | 0.41368 | 0.42748 | 0.40350 | 0.42653 | 0.40273 | 0.41399 | 0.41524 | 0.50042 |
| Sub#9 | 2.59 | 0.35291 | 0.38944 | 0.35553 | 0.40094 | 0.36619 | 0.39963 | 0.38201 | 0.40371 | 0.34289 | 0.45076 |
| Sub#10 | 3.21 | 0.36666 | 0.38532 | 0.36881 | 0.38825 | 0.38479 | 0.40465 | 0.39886 | 0.41071 | 0.39406 | 0.49141 |
| Sub#11 | 3.36 | 0.37502 | 0.39636 | 0.37134 | 0.38957 | 0.37833 | 0.39632 | 0.38559 | 0.39775 | 0.39316 | 0.46799 |
| Sub#12 | 3.58 | 0.39121 | 0.40463 | 0.38346 | 0.39655 | 0.39634 | 0.41382 | 0.42072 | 0.43032 | 0.44778 | 0.53463 |
| Sub#13 | 3.84 | 0.38977 | 0.41000 | 0.36912 | 0.38945 | 0.36693 | 0.40710 | 0.37893 | 0.39553 | 0.39453 | 0.48797 |
| Sub#14 | 4.02 | 0.36545 | 0.39557 | 0.33244 | 0.36198 | 0.38623 | 0.41607 | 0.35860 | 0.38019 | 0.33231 | 0.49355 |
| Sub#15 | 4.25 | 0.39306 | 0.40810 | 0.38012 | 0.39869 | 0.37602 | 0.39337 | 0.41455 | 0.42854 | 0.37625 | 0.53048 |
| Sub#16 | 4.39 | 0.39611 | 0.40994 | 0.38552 | 0.39935 | 0.43344 | 0.44946 | 0.39304 | 0.40732 | 0.37793 | 0.47170 |
| Sub#17 | 4.72 | 0.41527 | 0.43013 | 0.39371 | 0.40531 | 0.41920 | 0.43067 | 0.39365 | 0.40563 | 0.39961 | 0.51589 |
| Sub#18 | 5.02 | 0.42049 | 0.43441 | 0.39287 | 0.40973 | 0.43023 | 0.44590 | 0.40630 | 0.41931 | 0.41618 | 0.51131 |
| Sub#19 | 5.58 | 0.41980 | 0.43250 | 0.37608 | 0.39087 | 0.42793 | 0.43681 | 0.44952 | 0.45417 | 0.39163 | 0.49749 |
| Sub#20 | 5.75 | 0.41643 | 0.42884 | 0.40444 | 0.41691 | 0.39296 | 0.41253 | 0.36903 | 0.38107 | 0.40070 | 0.48933 |
| Sub#21 | 6.08 | 0.43363 | 0.44346 | 0.41297 | 0.42453 | 0.43614 | 0.44690 | 0.43869 | 0.44531 | 0.42941 | 0.51760 |
| Sub#22 | 6.42 | 0.42079 | 0.43563 | 0.39563 | 0.41001 | 0.39908 | 0.40762 | 0.43852 | 0.44146 | 0.39572 | 0.49590 |
| Sub#23 | 6.83 | 0.38846 | 0.40975 | 0.37132 | 0.38703 | 0.38581 | 0.41261 | 0.41008 | 0.42747 | 0.39750 | 0.49430 |
| Sub#24 | 7.00 | 0.40871 | 0.41826 | 0.39311 | 0.40231 | 0.42167 | 0.42968 | 0.42985 | 0.43871 | 0.39928 | 0.49270 |
| Sub#25 | 7.33 | 0.40671 | 0.41662 | 0.40665 | 0.41599 | 0.40142 | 0.41081 | 0.42005 | 0.43503 | 0.41992 | 0.50786 |
| Sub#26 | 7.50 | 0.42354 | 0.43144 | 0.40055 | 0.40739 | 0.42513 | 0.43546 | 0.40790 | 0.41292 | 0.41247 | 0.49083 |
| Sub#27 | 7.75 | 0.43164 | 0.43880 | 0.40735 | 0.41337 | 0.41495 | 0.41921 | 0.41569 | 0.42337 | 0.40128 | 0.49360 |
| Sub#28 | 8.08 | 0.42642 | 0.43408 | 0.40925 | 0.41466 | 0.38875 | 0.40081 | 0.40352 | 0.41395 | 0.35365 | 0.49741 |
| Sub#29 | 8.58 | 0.44200 | 0.44833 | 0.42004 | 0.42918 | 0.43521 | 0.44107 | 0.42403 | 0.42864 | 0.38784 | 0.50899 |
| Sub#30 | 8.75 | 0.39559 | 0.40174 | 0.39102 | 0.39963 | 0.40293 | 0.40766 | 0.41894 | 0.42002 | 0.40327 | 0.47793 |
| Sub#31 | 8.83 | 0.40872 | 0.41925 | 0.40459 | 0.41461 | 0.40160 | 0.41274 | 0.41985 | 0.42705 | 0.40183 | 0.48177 |
| Sub#32 | 9.17 | 0.40633 | 0.41281 | 0.40652 | 0.40794 | 0.39679 | 0.40244 | 0.40319 | 0.41753 | 0.41993 | 0.49381 |
| Sub#33 | 9.25 | 0.43047 | 0.43824 | 0.40725 | 0.41799 | 0.40350 | 0.41394 | 0.42427 | 0.43496 | 0.38732 | 0.49418 |
| Sub#34 | 9.50 | 0.43261 | 0.43940 | 0.42414 | 0.42736 | 0.42320 | 0.42516 | 0.41166 | 0.42437 | 0.40515 | 0.48074 |
| Sub#35 | 9.75 | 0.40262 | 0.40925 | 0.38899 | 0.40039 | 0.39973 | 0.40697 | 0.42052 | 0.42100 | 0.41276 | 0.49675 |
| Sub#36 | 10.08 | 0.41414 | 0.42911 | 0.41368 | 0.42748 | 0.40350 | 0.42653 | 0.40273 | 0.41399 | 0.41524 | 0.50042 |
| Sub#37 | 10.58 | 0.42549 | 0.43185 | 0.40577 | 0.41154 | 0.40941 | 0.41756 | 0.41897 | 0.42764 | 0.43022 | 0.51962 |
| Sub#38 | 10.83 | 0.42184 | 0.43025 | 0.41474 | 0.42419 | 0.42223 | 0.43582 | 0.42245 | 0.42749 | 0.43857 | 0.52091 |
| Sub#39 | 11.33 | 0.42718 | 0.43613 | 0.40723 | 0.41599 | 0.45449 | 0.46012 | 0.42245 | 0.42661 | 0.41204 | 0.53987 |
| Sub#40 | 11.75 | 0.44447 | 0.45039 | 0.41399 | 0.41929 | 0.42915 | 0.43526 | 0.44354 | 0.44862 | 0.43714 | 0.49678 |
| Sub#41 | 12 | 0.45087 | 0.45409 | 0.42143 | 0.42776 | 0.42062 | 0.42925 | 0.42773 | 0.43343 | 0.44745 | 0.51958 |
| Sub#42 | 12 | 0.42691 | 0.43250 | 0.39850 | 0.40259 | 0.41301 | 0.41765 | 0.41097 | 0.40865 | 0.38248 | 0.45840 |
| Sub#43 | 12 | 0.40680 | 0.41569 | 0.38614 | 0.40005 | 0.39722 | 0.40569 | 0.39103 | 0.39651 | 0.38487 | 0.46453 |
| Sub#44 | 12.25 | 0.44544 | 0.45205 | 0.41357 | 0.42624 | 0.42818 | 0.43684 | 0.44261 | 0.44762 | 0.37914 | 0.50997 |
| Sub#45 | 13 | 0.39384 | 0.39740 | 0.38792 | 0.39186 | 0.37753 | 0.38663 | 0.40875 | 0.40579 | 0.38543 | 0.46356 |
| Sub#46 | 14.08 | 0.43573 | 0.44144 | 0.43265 | 0.44221 | 0.41262 | 0.42458 | 0.42619 | 0.43539 | 0.42227 | 0.49713 |
| Sub#47 | 15 | 0.40683 | 0.41737 | 0.37628 | 0.38399 | 0.38785 | 0.41310 | 0.40252 | 0.41565 | 0.39672 | 0.47989 |
| Sub#48 | 15 | 0.40053 | 0.40993 | 0.37243 | 0.38522 | 0.43236 | 0.44278 | 0.34304 | 0.35698 | 0.38210 | 0.51548 |
| Sub#49 | 15.83 | 0.45641 | 0.45856 | 0.42228 | 0.42760 | 0.39938 | 0.40993 | 0.43885 | 0.44316 | 0.42163 | 0.50583 |
| Sub#50 | 16 | 0.40244 | 0.41249 | 0.37468 | 0.38228 | 0.40197 | 0.42085 | 0.38811 | 0.38995 | 0.38727 | 0.51741 |
| Sub#51 | 16 | 0.42831 | 0.43091 | 0.39707 | 0.40269 | 0.39483 | 0.40405 | 0.38232 | 0.39079 | 0.37266 | 0.45162 |
| Sub#52 | 17 | 0.43708 | 0.43844 | 0.42258 | 0.42767 | 0.41524 | 0.42203 | 0.39820 | 0.39827 | 0.43814 | 0.51511 |
| Sub#53 | 17 | 0.41312 | 0.42409 | 0.37332 | 0.38157 | 0.41598 | 0.43468 | 0.43023 | 0.43385 | 0.39526 | 0.50621 |
| Sub#54 | 17 | 0.42712 | 0.43767 | 0.39571 | 0.40157 | 0.40669 | 0.41580 | 0.38878 | 0.38740 | 0.39921 | 0.53754 |
| Sub#55 | 18 | 0.46144 | 0.45999 | 0.42946 | 0.42930 | 0.42151 | 0.41678 | 0.39883 | 0.40371 | 0.41130 | 0.48096 |
| Sub#56 | 18 | 0.47285 | 0.47378 | 0.42933 | 0.43332 | 0.42578 | 0.43261 | 0.43091 | 0.43288 | 0.41488 | 0.47516 |
| Sub#57 | 19 | 0.47195 | 0.47149 | 0.43832 | 0.44322 | 0.39760 | 0.39807 | 0.42136 | 0.42008 | 0.40503 | 0.45679 |
| Sub#58 | 20 | 0.46205 | 0.46079 | 0.44056 | 0.45279 | 0.45455 | 0.46608 | 0.43015 | 0.43280 | 0.41562 | 0.51010 |
| Sub#59 | 21 | 0.44765 | 0.45314 | 0.43755 | 0.44404 | 0.40125 | 0.39273 | 0.40783 | 0.40422 | 0.40788 | 0.48414 |
| Sub#60 | 22 | 0.44855 | 0.46017 | 0.44296 | 0.44890 | 0.41432 | 0.42651 | 0.41117 | 0.41572 | 0.42532 | 0.50674 |
| Sub#61 | 22 | 0.48175 | 0.47934 | 0.46340 | 0.47021 | 0.42820 | 0.43611 | 0.44965 | 0.44776 | 0.41914 | 0.47239 |
| Sub#62 | 23 | 0.48181 | 0.48365 | 0.43620 | 0.43394 | 0.48721 | 0.48223 | 0.41508 | 0.41745 | 0.44034 | 0.48985 |
| Sub#63 | 23 | 0.45678 | 0.45334 | 0.40852 | 0.41820 | 0.48202 | 0.48180 | 0.44617 | 0.44596 | 0.40421 | 0.50209 |
| Sub#64 | 24 | 0.40575 | 0.41593 | 0.36931 | 0.38041 | 0.43512 | 0.44863 | 0.43659 | 0.44026 | 0.39110 | 0.47818 |
| Sub#65 | 25 | 0.40825 | 0.41662 | 0.39567 | 0.40280 | 0.41468 | 0.42429 | 0.35734 | 0.37445 | 0.40821 | 0.49246 |

Supplemental Table 2: Mean diffusivity (MD) of each subject before and after free water elimination represented by UC (uncorrected) and C (corrected), respectively. The MD values for left (L) and right (R) cingulate gyrus part of cingulum (cgc), left and right cingulum hippocampal part (cgh) and fornix (fx) are shown. The unit of MD is 10^-3^ mm^2^/s. This table shows the quantitative data of Fig. 4.

| **Sub ID** | **Age (Years)** | **cgc-L** | | **cgc-R** | | **cgh-L** | | **cgh-R** | | **fx** | |
| --- | --- | --- | --- | --- | --- | --- | --- | --- | --- | --- | --- |
|  |  | **MD (UC)** | **MD (C)** | **MD (UC)** | **MD (C)** | **MD (UC)** | **MD (C)** | **MD (UC)** | **MD (C)** | **MD (UC)** | **MD (C)** |
| Sub#1 | 0.02 | 1.25352 | 0.86463 | 1.24577 | 0.85498 | 1.24623 | 0.78540 | 1.20870 | 0.83313 | 1.27779 | 0.86261 |
| Sub#2 | 0.03 | 1.21506 | 0.89120 | 1.20154 | 0.91472 | 1.17966 | 0.82281 | 1.15914 | 0.84178 | 1.22325 | 0.82625 |
| Sub#3 | 0.05 | 1.17661 | 0.91777 | 1.15732 | 0.97446 | 1.11308 | 0.86023 | 1.10958 | 0.85043 | 1.16870 | 0.78990 |
| Sub#4 | 0.42 | 0.91967 | 0.74423 | 0.95255 | 0.81234 | 0.92876 | 0.71296 | 0.93005 | 0.78943 | 1.20959 | 0.75496 |
| Sub#5 | 1.99 | 0.86271 | 0.69813 | 0.87499 | 0.74419 | 0.87551 | 0.71993 | 0.87811 | 0.74396 | 1.20713 | 0.75238 |
| Sub#6 | 2.17 | 0.80370 | 0.67135 | 0.79426 | 0.71748 | 0.80095 | 0.66905 | 0.80453 | 0.66195 | 1.20467 | 0.74980 |
| Sub#7 | 2.35 | 0.89000 | 0.70758 | 0.88075 | 0.76598 | 0.84776 | 0.71636 | 0.82087 | 0.73597 | 1.23749 | 0.67272 |
| Sub#8 | 2.50 | 0.81577 | 0.68718 | 0.84476 | 0.70914 | 0.81696 | 0.65128 | 0.85670 | 0.68993 | 1.31531 | 0.83700 |
| Sub#9 | 2.59 | 0.94035 | 0.75949 | 0.93779 | 0.68252 | 0.88533 | 0.71104 | 0.86042 | 0.73753 | 1.51369 | 1.00262 |
| Sub#10 | 3.21 | 0.83235 | 0.71375 | 0.84067 | 0.73311 | 0.86608 | 0.69239 | 0.84143 | 0.73400 | 1.28169 | 0.76497 |
| Sub#11 | 3.36 | 0.84900 | 0.69257 | 0.83797 | 0.71588 | 0.86258 | 0.69585 | 0.88189 | 0.73879 | 1.20308 | 0.78380 |
| Sub#12 | 3.58 | 0.77734 | 0.67455 | 0.76781 | 0.69097 | 0.81714 | 0.67955 | 0.81877 | 0.68276 | 1.17814 | 0.66392 |
| Sub#13 | 3.84 | 0.87828 | 0.72093 | 0.86244 | 0.73955 | 0.91556 | 0.67575 | 0.87055 | 0.71782 | 1.25672 | 0.77880 |
| Sub#14 | 4.02 | 0.88496 | 0.69895 | 0.87975 | 0.75719 | 0.91022 | 0.64959 | 0.88773 | 0.72649 | 1.54047 | 0.89976 |
| Sub#15 | 4.25 | 0.83222 | 0.70934 | 0.85583 | 0.72377 | 0.83742 | 0.70086 | 0.88138 | 0.74218 | 1.53521 | 0.81409 |
| Sub#16 | 4.39 | 0.80908 | 0.68637 | 0.82139 | 0.72396 | 0.83491 | 0.65767 | 0.85925 | 0.71972 | 1.36670 | 0.93035 |
| Sub#17 | 4.72 | 0.80387 | 0.66794 | 0.79573 | 0.70082 | 0.80809 | 0.65403 | 0.81939 | 0.68727 | 1.27996 | 0.67919 |
| Sub#18 | 5.02 | 0.80141 | 0.67414 | 0.79541 | 0.67941 | 0.83634 | 0.65852 | 0.81633 | 0.71977 | 1.27170 | 0.74951 |
| Sub#19 | 5.58 | 0.77545 | 0.64305 | 0.80335 | 0.68629 | 0.78683 | 0.67980 | 0.79569 | 0.68542 | 1.41272 | 0.93110 |
| Sub#20 | 5.75 | 0.79040 | 0.64687 | 0.79613 | 0.69348 | 0.82647 | 0.64163 | 0.85716 | 0.72984 | 1.23875 | 0.77610 |
| Sub#21 | 6.08 | 0.76354 | 0.65178 | 0.81414 | 0.70219 | 0.80161 | 0.70146 | 0.82174 | 0.68541 | 1.26777 | 0.81187 |
| Sub#22 | 6.42 | 0.79830 | 0.66858 | 0.78901 | 0.68213 | 0.82145 | 0.68768 | 0.80305 | 0.69661 | 1.36854 | 0.89145 |
| Sub#23 | 6.83 | 0.85183 | 0.68355 | 0.84717 | 0.72309 | 0.89825 | 0.65177 | 0.86107 | 0.70557 | 1.33408 | 0.85524 |
| Sub#24 | 7.00 | 0.77246 | 0.66254 | 0.78561 | 0.68617 | 0.79070 | 0.68500 | 0.81023 | 0.71239 | 1.29961 | 0.81902 |
| Sub#25 | 7.33 | 0.78697 | 0.66807 | 0.77275 | 0.66457 | 0.81583 | 0.64772 | 0.82042 | 0.68600 | 1.31786 | 0.84751 |
| Sub#26 | 7.50 | 0.78276 | 0.65993 | 0.76238 | 0.68893 | 0.79794 | 0.66547 | 0.79423 | 0.69879 | 1.14397 | 0.69954 |
| Sub#27 | 7.75 | 0.77133 | 0.66611 | 0.78936 | 0.70212 | 0.76830 | 0.62996 | 0.80678 | 0.68650 | 1.35900 | 0.90223 |
| Sub#28 | 8.08 | 0.75250 | 0.65315 | 0.74155 | 0.66810 | 0.78131 | 0.65758 | 0.81402 | 0.66763 | 1.41135 | 0.78979 |
| Sub#29 | 8.58 | 0.73506 | 0.63390 | 0.73369 | 0.63899 | 0.78055 | 0.60959 | 0.78246 | 0.67412 | 1.36816 | 0.80581 |
| Sub#30 | 8.75 | 0.74682 | 0.62721 | 0.74186 | 0.68388 | 0.74147 | 0.65645 | 0.77682 | 0.67400 | 1.13279 | 0.69811 |
| Sub#31 | 8.83 | 0.77963 | 0.67683 | 0.77588 | 0.68908 | 0.83049 | 0.66714 | 0.81853 | 0.68190 | 1.23581 | 0.78972 |
| Sub#32 | 9.17 | 0.73992 | 0.65956 | 0.73012 | 0.65867 | 0.76065 | 0.67967 | 0.78273 | 0.65742 | 1.07988 | 0.64129 |
| Sub#33 | 9.25 | 0.75488 | 0.65612 | 0.76389 | 0.66516 | 0.80094 | 0.64672 | 0.81837 | 0.68194 | 1.36751 | 0.84622 |
| Sub#34 | 9.50 | 0.74677 | 0.63848 | 0.72843 | 0.63374 | 0.75933 | 0.64938 | 0.80436 | 0.65283 | 1.15182 | 0.69975 |
| Sub#35 | 9.75 | 0.76345 | 0.66713 | 0.74878 | 0.65942 | 0.75984 | 0.53350 | 0.76303 | 0.64433 | 1.26144 | 0.80547 |
| Sub#36 | 10.08 | 0.81577 | 0.68718 | 0.84476 | 0.70914 | 0.81696 | 0.65128 | 0.85670 | 0.68993 | 1.31531 | 0.83700 |
| Sub#37 | 10.58 | 0.73122 | 0.64306 | 0.74151 | 0.65883 | 0.77755 | 0.61534 | 0.78493 | 0.68849 | 1.17993 | 0.69809 |
| Sub#38 | 10.83 | 0.75925 | 0.63954 | 0.75823 | 0.66772 | 0.82075 | 0.67972 | 0.80050 | 0.68576 | 1.20701 | 0.73661 |
| Sub#39 | 11.33 | 0.77016 | 0.66299 | 0.77770 | 0.67739 | 0.78458 | 0.64989 | 0.78575 | 0.67740 | 1.25322 | 0.59526 |
| Sub#40 | 11.75 | 0.75663 | 0.64502 | 0.73756 | 0.66121 | 0.78247 | 0.63967 | 0.78470 | 0.65856 | 1.15094 | 0.76295 |
| Sub#41 | 12 | 0.76123 | 0.63389 | 0.75858 | 0.65484 | 0.77990 | 0.65739 | 0.81166 | 0.67237 | 1.14731 | 0.70217 |
| Sub#42 | 12 | 0.70649 | 0.63280 | 0.70091 | 0.64060 | 0.73690 | 0.67394 | 0.69790 | 0.64609 | 1.07475 | 0.64740 |
| Sub#43 | 12 | 0.76219 | 0.67058 | 0.73818 | 0.64829 | 0.72103 | 0.67076 | 0.73408 | 0.66914 | 1.10483 | 0.68098 |
| Sub#44 | 12.25 | 0.74208 | 0.62889 | 0.77707 | 0.66859 | 0.76453 | 0.61751 | 0.81465 | 0.64916 | 1.50014 | 0.95645 |
| Sub#45 | 13 | 0.70647 | 0.62889 | 0.71328 | 0.64277 | 0.74675 | 0.65903 | 0.71236 | 0.61371 | 1.13908 | 0.71398 |
| Sub#46 | 14.08 | 0.77043 | 0.64734 | 0.76816 | 0.65316 | 0.81452 | 0.65972 | 0.84857 | 0.65966 | 1.16382 | 0.70411 |
| Sub#47 | 15 | 0.74624 | 0.65221 | 0.74728 | 0.67322 | 0.81871 | 0.62240 | 0.79332 | 0.65684 | 1.17710 | 0.69768 |
| Sub#48 | 15 | 0.75312 | 0.64985 | 0.75760 | 0.66487 | 0.73511 | 0.62659 | 0.75234 | 0.67181 | 1.27528 | 0.64642 |
| Sub#49 | 15.83 | 0.74474 | 0.62809 | 0.75131 | 0.64552 | 0.77128 | 0.63265 | 0.77071 | 0.64472 | 1.16042 | 0.67021 |
| Sub#50 | 16 | 0.72633 | 0.62933 | 0.72022 | 0.67425 | 0.75266 | 0.61715 | 0.70938 | 0.64969 | 1.27039 | 0.64097 |
| Sub#51 | 16 | 0.68452 | 0.62040 | 0.67235 | 0.62052 | 0.70175 | 0.65427 | 0.69194 | 0.62414 | 1.09485 | 0.68132 |
| Sub#52 | 17 | 0.70504 | 0.61522 | 0.70562 | 0.63257 | 0.72670 | 0.64431 | 0.72316 | 0.64733 | 1.10029 | 0.62306 |
| Sub#53 | 17 | 0.75942 | 0.67087 | 0.75556 | 0.68757 | 0.82376 | 0.68328 | 0.77996 | 0.69464 | 1.38425 | 0.87995 |
| Sub#54 | 17 | 0.70742 | 0.60348 | 0.70630 | 0.64775 | 0.77414 | 0.67562 | 0.72656 | 0.65665 | 1.31686 | 0.70827 |
| Sub#55 | 18 | 0.68114 | 0.60064 | 0.68657 | 0.62299 | 0.67587 | 0.60735 | 0.70295 | 0.63974 | 1.08470 | 0.69673 |
| Sub#56 | 18 | 0.72383 | 0.63904 | 0.73998 | 0.67823 | 0.75208 | 0.63200 | 0.72627 | 0.62418 | 1.01328 | 0.65234 |
| Sub#57 | 19 | 0.70231 | 0.57254 | 0.72740 | 0.58982 | 0.70198 | 0.61327 | 0.71318 | 0.59110 | 1.04167 | 0.72048 |
| Sub#58 | 20 | 0.64664 | 0.57899 | 0.66144 | 0.60279 | 0.67060 | 0.56577 | 0.68714 | 0.59033 | 1.16031 | 0.65670 |
| Sub#59 | 21 | 0.71145 | 0.59183 | 0.71307 | 0.59554 | 0.68013 | 0.53309 | 0.69681 | 0.59953 | 1.11010 | 0.68618 |
| Sub#60 | 22 | 0.71622 | 0.64246 | 0.70981 | 0.65035 | 0.77011 | 0.66429 | 0.73528 | 0.66367 | 1.22215 | 0.82343 |
| Sub#61 | 22 | 0.69660 | 0.57877 | 0.69255 | 0.62468 | 0.77697 | 0.62882 | 0.74914 | 0.59395 | 1.01451 | 0.66108 |
| Sub#62 | 23 | 0.64839 | 0.58367 | 0.66626 | 0.59337 | 0.73524 | 0.59495 | 0.76048 | 0.63382 | 0.96545 | 0.63730 |
| Sub#63 | 23 | 0.69205 | 0.58597 | 0.68880 | 0.58597 | 0.67254 | 0.61476 | 0.68718 | 0.59688 | 1.21975 | 0.74714 |
| Sub#64 | 24 | 0.77175 | 0.64685 | 0.70203 | 0.62445 | 0.78178 | 0.63742 | 0.79309 | 0.64279 | 1.34452 | 0.94091 |
| Sub#65 | 25 | 0.71260 | 0.62180 | 0.75549 | 0.68482 | 0.77856 | 0.63903 | 0.83784 | 0.65803 | 1.28334 | 0.88217 |

Supplemental Table 3: Axial diffusivity (AD) of each subject before and after free water elimination represented by UC (uncorrected) and C (corrected), respectively. The AD values for left (L) and right (R) cingulate gyrus part of cingulum (cgc), left and right cingulum hippocampal part (cgh) and fornix (fx) are shown. The unit of AD is 10^-3^ mm^2^/s. This table shows the quantitative data of Fig. 5.

| **Sub ID** | **Age (Years)** | **cgc-L** | | **cgc-R** | | **cgh-L** | | **cgh-R** | | **fx** | |
| --- | --- | --- | --- | --- | --- | --- | --- | --- | --- | --- | --- |
|  |  | **AD (UC)** | **AD (C)** | **AD (UC)** | **AD (C)** | **AD (UC)** | **AD (C)** | **AD (UC)** | **AD (C)** | **AD (UC)** | **AD (C)** |
| Sub#1 | 0.02 | 1.62663 | 1.15465 | 1.59002 | 1.14634 | 1.60917 | 1.06155 | 1.54216 | 1.10415 | 1.75985 | 1.22766 |
| Sub#2 | 0.03 | 1.58867 | 1.19574 | 1.54936 | 1.22145 | 1.56272 | 1.12906 | 1.52842 | 1.14212 | 1.73493 | 1.20776 |
| Sub#3 | 0.05 | 1.55071 | 1.23682 | 1.50871 | 1.29655 | 1.51627 | 1.19657 | 1.51468 | 1.18009 | 1.71000 | 1.18786 |
| Sub#4 | 0.42 | 1.30153 | 1.05139 | 1.31373 | 1.12990 | 1.33609 | 1.02980 | 1.31418 | 1.12036 | 1.71978 | 1.11240 |
| Sub#5 | 1.99 | 1.24458 | 1.00468 | 1.22823 | 1.05159 | 1.26062 | 1.03436 | 1.29367 | 1.09400 | 1.72503 | 1.11816 |
| Sub#6 | 2.17 | 1.17178 | 0.97748 | 1.12373 | 1.02108 | 1.17803 | 0.98563 | 1.19402 | 0.97862 | 1.73028 | 1.12392 |
| Sub#7 | 2.35 | 1.25192 | 0.99785 | 1.20892 | 1.05843 | 1.23846 | 1.03879 | 1.17979 | 1.06152 | 1.80573 | 1.03971 |
| Sub#8 | 2.50 | 1.20350 | 1.01172 | 1.24808 | 1.04746 | 1.19870 | 0.96137 | 1.25897 | 1.00076 | 1.92149 | 1.26727 |
| Sub#9 | 2.59 | 1.31134 | 1.06815 | 1.31434 | 0.96977 | 1.25660 | 1.01585 | 1.23759 | 1.06757 | 2.08723 | 1.43865 |
| Sub#10 | 3.21 | 1.17385 | 1.00873 | 1.18506 | 1.03920 | 1.25278 | 0.99681 | 1.23054 | 1.07458 | 1.85201 | 1.14274 |
| Sub#11 | 3.36 | 1.21409 | 0.98819 | 1.18834 | 1.01409 | 1.23776 | 0.98982 | 1.27024 | 1.06354 | 1.74246 | 1.16594 |
| Sub#12 | 3.58 | 1.12495 | 0.97562 | 1.09646 | 0.99063 | 1.19059 | 0.99002 | 1.22233 | 1.00837 | 1.79257 | 1.04872 |
| Sub#13 | 3.84 | 1.27002 | 1.03833 | 1.21860 | 1.04868 | 1.29898 | 0.96193 | 1.25461 | 1.02337 | 1.82286 | 1.16912 |
| Sub#14 | 4.02 | 1.25147 | 0.98974 | 1.19876 | 1.04619 | 1.32501 | 0.94353 | 1.25184 | 1.03001 | 2.11522 | 1.33179 |
| Sub#15 | 4.25 | 1.19959 | 1.02425 | 1.21737 | 1.03169 | 1.19995 | 1.00203 | 1.30530 | 1.09571 | 2.17935 | 1.21308 |
| Sub#16 | 4.39 | 1.17342 | 0.99254 | 1.17456 | 1.03851 | 1.26564 | 0.98795 | 1.24654 | 1.04254 | 1.94648 | 1.37103 |
| Sub#17 | 4.72 | 1.19306 | 0.98749 | 1.15189 | 1.01494 | 1.21240 | 0.97553 | 1.19333 | 0.99855 | 1.85712 | 1.05229 |
| Sub#18 | 5.02 | 1.19046 | 0.99831 | 1.15177 | 0.98768 | 1.27059 | 0.99757 | 1.20167 | 1.05944 | 1.87134 | 1.14005 |
| Sub#19 | 5.58 | 1.15327 | 0.95336 | 1.14433 | 0.97976 | 1.18380 | 1.02532 | 1.22445 | 1.05077 | 2.01793 | 1.38095 |
| Sub#20 | 5.75 | 1.17542 | 0.95673 | 1.16651 | 1.01595 | 1.20503 | 0.93154 | 1.21682 | 1.03821 | 1.80057 | 1.15939 |
| Sub#21 | 6.08 | 1.14502 | 0.97751 | 1.19604 | 1.03052 | 1.21553 | 1.06495 | 1.24615 | 1.03130 | 1.88878 | 1.24660 |
| Sub#22 | 6.42 | 1.18559 | 0.99409 | 1.14571 | 0.99323 | 1.19673 | 0.99796 | 1.22412 | 1.05454 | 1.97740 | 1.32369 |
| Sub#23 | 6.83 | 1.23242 | 0.98849 | 1.19957 | 1.02295 | 1.30233 | 0.94960 | 1.27391 | 1.04373 | 1.93054 | 1.27351 |
| Sub#24 | 7.00 | 1.13748 | 0.97141 | 1.13533 | 0.99008 | 1.18228 | 1.02841 | 1.21816 | 1.06777 | 1.88368 | 1.22332 |
| Sub#25 | 7.33 | 1.15629 | 0.97566 | 1.13664 | 0.97442 | 1.19319 | 0.93780 | 1.22662 | 1.02270 | 1.93674 | 1.29138 |
| Sub#26 | 7.50 | 1.16621 | 0.97749 | 1.10701 | 0.99895 | 1.19908 | 0.99844 | 1.16871 | 1.02816 | 1.68214 | 1.05634 |
| Sub#27 | 7.75 | 1.15993 | 0.99459 | 1.14687 | 1.01874 | 1.14141 | 0.93115 | 1.20183 | 1.01829 | 1.97211 | 1.34662 |
| Sub#28 | 8.08 | 1.12583 | 0.97517 | 1.09057 | 0.98036 | 1.13014 | 0.94878 | 1.19541 | 0.97634 | 1.96912 | 1.16814 |
| Sub#29 | 8.58 | 1.11590 | 0.95707 | 1.08942 | 0.95075 | 1.17962 | 0.90549 | 1.16983 | 1.00567 | 1.96056 | 1.21347 |
| Sub#30 | 8.75 | 1.08968 | 0.91040 | 1.06597 | 0.98537 | 1.08688 | 0.96009 | 1.16092 | 1.00205 | 1.65277 | 1.04335 |
| Sub#31 | 8.83 | 1.14726 | 0.99565 | 1.13089 | 1.00555 | 1.21365 | 0.96862 | 1.22151 | 1.00734 | 1.79902 | 1.18996 |
| Sub#32 | 9.17 | 1.08847 | 0.96698 | 1.06683 | 0.95893 | 1.11232 | 0.99158 | 1.14961 | 0.96851 | 1.59848 | 0.97052 |
| Sub#33 | 9.25 | 1.13746 | 0.98669 | 1.11887 | 0.97379 | 1.18460 | 0.95099 | 1.23130 | 1.02213 | 1.95288 | 1.25512 |
| Sub#34 | 9.50 | 1.12462 | 0.95851 | 1.08536 | 0.93904 | 1.13565 | 0.97134 | 1.19305 | 0.96962 | 1.67623 | 1.04518 |
| Sub#35 | 9.75 | 1.12053 | 0.97827 | 1.07871 | 0.95237 | 1.11568 | 0.76882 | 1.14676 | 0.96416 | 1.83945 | 1.21688 |
| Sub#36 | 10.08 | 1.20350 | 1.01172 | 1.24808 | 1.04746 | 1.19870 | 0.96137 | 1.25897 | 1.00076 | 1.92149 | 1.26727 |
| Sub#37 | 10.58 | 1.09257 | 0.95928 | 1.08509 | 0.96383 | 1.14582 | 0.89505 | 1.16559 | 1.02244 | 1.75628 | 1.08777 |
| Sub#38 | 10.83 | 1.13489 | 0.94785 | 1.11788 | 0.98378 | 1.22378 | 1.01123 | 1.19678 | 1.01676 | 1.80341 | 1.12982 |
| Sub#39 | 11.33 | 1.15354 | 0.99204 | 1.13867 | 0.98783 | 1.21457 | 0.99669 | 1.17921 | 1.01041 | 1.84620 | 0.92805 |
| Sub#40 | 11.75 | 1.14910 | 0.97184 | 1.08753 | 0.97501 | 1.16911 | 0.94664 | 1.20434 | 1.00213 | 1.72951 | 1.16778 |
| Sub#41 | 12 | 1.17634 | 0.96917 | 1.13210 | 0.97345 | 1.16659 | 0.97527 | 1.22040 | 1.00137 | 1.74235 | 1.08849 |
| Sub#42 | 12 | 1.06400 | 0.95090 | 1.02164 | 0.93544 | 1.08974 | 0.99495 | 1.03173 | 0.95235 | 1.54587 | 0.96661 |
| Sub#43 | 12 | 1.12065 | 0.98344 | 1.06151 | 0.93923 | 1.04690 | 0.97858 | 1.06746 | 0.97475 | 1.59735 | 1.00845 |
| Sub#44 | 12.25 | 1.12897 | 0.95352 | 1.14654 | 0.98846 | 1.14425 | 0.92126 | 1.24847 | 0.98766 | 2.14203 | 1.44401 |
| Sub#45 | 13 | 1.02881 | 0.91550 | 1.03173 | 0.92920 | 1.06623 | 0.94411 | 1.05758 | 0.90156 | 1.63519 | 1.05364 |
| Sub#46 | 14.08 | 1.16646 | 0.97037 | 1.15365 | 0.97899 | 1.20589 | 0.97660 | 1.27304 | 0.97934 | 1.73676 | 1.06413 |
| Sub#47 | 15 | 1.09882 | 0.95626 | 1.06487 | 0.95841 | 1.18735 | 0.90878 | 1.16822 | 0.96450 | 1.71455 | 1.04186 |
| Sub#48 | 15 | 1.09705 | 0.94349 | 1.07849 | 0.94917 | 1.10864 | 0.94443 | 1.03973 | 0.93110 | 1.83887 | 0.97668 |
| Sub#49 | 15.83 | 1.15283 | 0.95714 | 1.12100 | 0.95425 | 1.13236 | 0.93407 | 1.17076 | 0.96913 | 1.71698 | 1.01905 |
| Sub#50 | 16 | 1.06553 | 0.92246 | 1.02446 | 0.96169 | 1.09776 | 0.90468 | 1.02572 | 0.93665 | 1.82992 | 0.97992 |
| Sub#51 | 16 | 1.02762 | 0.93224 | 0.97802 | 0.90647 | 1.01408 | 0.94852 | 0.98482 | 0.89351 | 1.55241 | 1.00219 |
| Sub#52 | 17 | 1.07331 | 0.92356 | 1.05728 | 0.94353 | 1.07625 | 0.94932 | 1.05555 | 0.94236 | 1.67022 | 0.96476 |
| Sub#53 | 17 | 1.12069 | 0.99181 | 1.07353 | 0.97807 | 1.21871 | 1.01843 | 1.16870 | 1.04025 | 1.99731 | 1.29733 |
| Sub#54 | 17 | 1.05999 | 0.89576 | 1.02289 | 0.93962 | 1.13034 | 0.98324 | 1.04641 | 0.94382 | 1.90136 | 1.07251 |
| Sub#55 | 18 | 1.06399 | 0.93050 | 1.03328 | 0.93108 | 1.01463 | 0.90109 | 1.02956 | 0.93443 | 1.58784 | 1.04367 |
| Sub#56 | 18 | 1.14060 | 0.99580 | 1.11344 | 1.01151 | 1.13033 | 0.94841 | 1.09803 | 0.93591 | 1.50309 | 0.98470 |
| Sub#57 | 19 | 1.10631 | 0.89030 | 1.10588 | 0.88729 | 1.02112 | 0.89059 | 1.06618 | 0.87743 | 1.51505 | 1.06240 |
| Sub#58 | 20 | 1.00350 | 0.89429 | 1.00073 | 0.91884 | 1.02102 | 0.87025 | 1.02849 | 0.88313 | 1.70197 | 0.99612 |
| Sub#59 | 21 | 1.08719 | 0.89792 | 1.07574 | 0.89954 | 0.99314 | 0.76352 | 1.02487 | 0.87737 | 1.61668 | 1.01862 |
| Sub#60 | 22 | 1.09301 | 0.98309 | 1.07946 | 0.98640 | 1.13966 | 0.98307 | 1.08599 | 0.97582 | 1.81200 | 1.27039 |
| Sub#61 | 22 | 1.10332 | 0.89959 | 1.07338 | 0.97040 | 1.15997 | 0.93370 | 1.15420 | 0.89539 | 1.50347 | 0.98900 |
| Sub#62 | 23 | 1.01658 | 0.91246 | 1.00218 | 0.88886 | 1.18032 | 0.93771 | 1.12730 | 0.93597 | 1.45238 | 0.97205 |
| Sub#63 | 23 | 1.07359 | 0.89436 | 1.01217 | 0.86407 | 1.04267 | 0.95550 | 1.05148 | 0.91006 | 1.77086 | 1.13154 |
| Sub#64 | 24 | 1.13853 | 0.94698 | 1.06922 | 0.97273 | 1.17928 | 0.96496 | 1.20550 | 0.96234 | 1.93136 | 1.39425 |
| Sub#65 | 25 | 1.05171 | 0.91460 | 1.01943 | 0.90873 | 1.16381 | 0.94591 | 1.17276 | 0.91841 | 1.87168 | 1.33232 |

Supplemental Table 4: Radial diffusivity (RD) of each subject before and after free water elimination represented by UC (uncorrected) and C (corrected), respectively. The RD values for left (L) and right (R) cingulate gyrus part of cingulum (cgc), left and right cingulum hippocampal part (cgh) and fornix (fx) are shown. The unit of RD is 10^-3^ mm^2^/s. This table shows the quantitative data of Fig. 6.

| **Sub ID** | **Age (Years)** | **cgc-L** | | **cgc-R** | | **cgh-L** | | **cgh-R** | | **fx** | |
| --- | --- | --- | --- | --- | --- | --- | --- | --- | --- | --- | --- |
|  |  | **RD (UC)** | **RD (C)** | **RD (UC)** | **RD (C)** | **RD (UC)** | **RD (C)** | **RD (UC)** | **RD (C)** | **RD (UC)** | **RD (C)** |
| Sub#1 | 0.02 | 1.06697 | 0.71962 | 1.07365 | 0.70930 | 1.06476 | 0.64733 | 1.04197 | 0.69761 | 1.03676 | 0.68008 |
| Sub#2 | 0.03 | 1.02826 | 0.73893 | 1.02764 | 0.76135 | 0.98813 | 0.66969 | 0.97451 | 0.69161 | 0.96741 | 0.63550 |
| Sub#3 | 0.05 | 0.98955 | 0.75824 | 0.98162 | 0.81341 | 0.91149 | 0.69206 | 0.90704 | 0.68560 | 0.89805 | 0.59091 |
| Sub#4 | 0.42 | 0.72873 | 0.59065 | 0.77196 | 0.65356 | 0.72510 | 0.55454 | 0.73799 | 0.62396 | 0.95450 | 0.57624 |
| Sub#5 | 1.99 | 0.67177 | 0.54485 | 0.69837 | 0.59049 | 0.68295 | 0.56271 | 0.67033 | 0.56894 | 0.94818 | 0.56949 |
| Sub#6 | 2.17 | 0.61965 | 0.51829 | 0.62952 | 0.56568 | 0.61241 | 0.51076 | 0.60978 | 0.50362 | 0.94186 | 0.56274 |
| Sub#7 | 2.35 | 0.70904 | 0.56245 | 0.71666 | 0.61975 | 0.65241 | 0.55514 | 0.64141 | 0.57320 | 0.95337 | 0.48922 |
| Sub#8 | 2.50 | 0.62190 | 0.52491 | 0.64310 | 0.53999 | 0.62609 | 0.49624 | 0.65557 | 0.53452 | 1.01222 | 0.62186 |
| Sub#9 | 2.59 | 0.75486 | 0.60515 | 0.74952 | 0.53890 | 0.69969 | 0.55863 | 0.67183 | 0.57251 | 1.22692 | 0.78461 |
| Sub#10 | 3.21 | 0.66161 | 0.56626 | 0.66847 | 0.58007 | 0.67273 | 0.54017 | 0.64688 | 0.56371 | 0.99653 | 0.57609 |
| Sub#11 | 3.36 | 0.66645 | 0.54476 | 0.66278 | 0.56677 | 0.67499 | 0.54887 | 0.68772 | 0.57641 | 0.93339 | 0.59273 |
| Sub#12 | 3.58 | 0.60353 | 0.52402 | 0.60348 | 0.54114 | 0.63042 | 0.52432 | 0.61699 | 0.51995 | 0.87093 | 0.47151 |
| Sub#13 | 3.84 | 0.68242 | 0.56224 | 0.68436 | 0.58499 | 0.72385 | 0.53267 | 0.67852 | 0.56505 | 0.97366 | 0.58365 |
| Sub#14 | 4.02 | 0.70170 | 0.55356 | 0.72024 | 0.61269 | 0.70283 | 0.50263 | 0.70568 | 0.57473 | 1.25310 | 0.68375 |
| Sub#15 | 4.25 | 0.64853 | 0.55188 | 0.67506 | 0.56980 | 0.65615 | 0.55027 | 0.66942 | 0.56542 | 1.21314 | 0.61460 |
| Sub#16 | 4.39 | 0.62691 | 0.53329 | 0.64481 | 0.56668 | 0.61955 | 0.49253 | 0.66561 | 0.55830 | 1.07680 | 0.71001 |
| Sub#17 | 4.72 | 0.60928 | 0.50817 | 0.61766 | 0.54376 | 0.60594 | 0.49328 | 0.63241 | 0.53163 | 0.99138 | 0.49264 |
| Sub#18 | 5.02 | 0.60689 | 0.51205 | 0.61724 | 0.52528 | 0.61922 | 0.48899 | 0.62367 | 0.54993 | 0.97187 | 0.55424 |
| Sub#19 | 5.58 | 0.58654 | 0.48790 | 0.63286 | 0.53955 | 0.58835 | 0.50705 | 0.58131 | 0.50274 | 1.11012 | 0.70617 |
| Sub#20 | 5.75 | 0.59789 | 0.49193 | 0.61095 | 0.53224 | 0.63718 | 0.49668 | 0.67732 | 0.57566 | 0.95784 | 0.58446 |
| Sub#21 | 6.08 | 0.57279 | 0.48892 | 0.62319 | 0.53802 | 0.59464 | 0.51972 | 0.60953 | 0.51247 | 0.95726 | 0.59450 |
| Sub#22 | 6.42 | 0.60465 | 0.50582 | 0.61066 | 0.52658 | 0.63381 | 0.53254 | 0.59251 | 0.51765 | 1.06412 | 0.67533 |
| Sub#23 | 6.83 | 0.66153 | 0.53108 | 0.67097 | 0.57316 | 0.69621 | 0.50286 | 0.65465 | 0.53650 | 1.03585 | 0.64610 |
| Sub#24 | 7.00 | 0.58995 | 0.50810 | 0.61076 | 0.53421 | 0.59490 | 0.51330 | 0.60627 | 0.53471 | 1.00758 | 0.61687 |
| Sub#25 | 7.33 | 0.60231 | 0.51427 | 0.59081 | 0.50965 | 0.62715 | 0.50268 | 0.61732 | 0.51765 | 1.00843 | 0.62557 |
| Sub#26 | 7.50 | 0.59103 | 0.50115 | 0.59007 | 0.53393 | 0.59737 | 0.49899 | 0.60699 | 0.53410 | 0.87488 | 0.52114 |
| Sub#27 | 7.75 | 0.57703 | 0.50186 | 0.61060 | 0.54381 | 0.58174 | 0.47937 | 0.60926 | 0.52061 | 1.05244 | 0.68004 |
| Sub#28 | 8.08 | 0.56583 | 0.49213 | 0.56703 | 0.51196 | 0.60690 | 0.51198 | 0.62333 | 0.51328 | 1.13246 | 0.60062 |
| Sub#29 | 8.58 | 0.54463 | 0.47232 | 0.55583 | 0.48311 | 0.58101 | 0.46164 | 0.58878 | 0.50835 | 1.07196 | 0.60199 |
| Sub#30 | 8.75 | 0.57539 | 0.48562 | 0.57980 | 0.53313 | 0.56876 | 0.50464 | 0.58477 | 0.50997 | 0.87280 | 0.52549 |
| Sub#31 | 8.83 | 0.59581 | 0.51742 | 0.59838 | 0.53085 | 0.63891 | 0.51639 | 0.61704 | 0.51918 | 0.95421 | 0.58959 |
| Sub#32 | 9.17 | 0.56564 | 0.50585 | 0.56177 | 0.50855 | 0.58481 | 0.52371 | 0.59930 | 0.50187 | 0.82058 | 0.47667 |
| Sub#33 | 9.25 | 0.56359 | 0.49083 | 0.58641 | 0.51084 | 0.60911 | 0.49459 | 0.61191 | 0.51185 | 1.07482 | 0.64177 |
| Sub#34 | 9.50 | 0.55785 | 0.47847 | 0.54996 | 0.48109 | 0.57117 | 0.48840 | 0.61002 | 0.49443 | 0.88962 | 0.52704 |
| Sub#35 | 9.75 | 0.58491 | 0.51155 | 0.58382 | 0.51295 | 0.58191 | 0.41584 | 0.57117 | 0.48442 | 0.97243 | 0.59976 |
| Sub#36 | 10.08 | 0.62190 | 0.52491 | 0.64310 | 0.53999 | 0.62609 | 0.49624 | 0.65557 | 0.53452 | 1.01222 | 0.62186 |
| Sub#37 | 10.58 | 0.55055 | 0.48494 | 0.56971 | 0.50633 | 0.59341 | 0.47548 | 0.59460 | 0.52151 | 0.89175 | 0.50325 |
| Sub#38 | 10.83 | 0.57142 | 0.48538 | 0.57840 | 0.50969 | 0.61924 | 0.51397 | 0.60236 | 0.52026 | 0.90881 | 0.54001 |
| Sub#39 | 11.33 | 0.57847 | 0.49846 | 0.59721 | 0.52216 | 0.56958 | 0.47650 | 0.58902 | 0.51089 | 0.95673 | 0.42887 |
| Sub#40 | 11.75 | 0.56040 | 0.48161 | 0.56258 | 0.50431 | 0.58916 | 0.48618 | 0.57488 | 0.48677 | 0.86165 | 0.56053 |
| Sub#41 | 12 | 0.55368 | 0.46625 | 0.57182 | 0.49554 | 0.58656 | 0.49845 | 0.60729 | 0.50786 | 0.84979 | 0.50900 |
| Sub#42 | 12 | 0.52773 | 0.47374 | 0.54055 | 0.49319 | 0.56047 | 0.51344 | 0.53098 | 0.49296 | 0.83919 | 0.48779 |
| Sub#43 | 12 | 0.58296 | 0.51415 | 0.57651 | 0.50282 | 0.55809 | 0.51686 | 0.56738 | 0.51633 | 0.85857 | 0.51724 |
| Sub#44 | 12.25 | 0.54863 | 0.46657 | 0.59234 | 0.50866 | 0.57467 | 0.46564 | 0.59775 | 0.47991 | 1.17919 | 0.71268 |
| Sub#45 | 13 | 0.54529 | 0.48558 | 0.55406 | 0.49955 | 0.58701 | 0.51650 | 0.53975 | 0.46978 | 0.89102 | 0.54415 |
| Sub#46 | 14.08 | 0.57241 | 0.48582 | 0.57541 | 0.49024 | 0.61884 | 0.50127 | 0.63634 | 0.49983 | 0.87736 | 0.52410 |
| Sub#47 | 15 | 0.56995 | 0.50018 | 0.58848 | 0.53062 | 0.63440 | 0.47921 | 0.60586 | 0.50301 | 0.90838 | 0.52559 |
| Sub#48 | 15 | 0.58116 | 0.50303 | 0.59716 | 0.52273 | 0.54834 | 0.46768 | 0.60865 | 0.54216 | 0.99349 | 0.48129 |
| Sub#49 | 15.83 | 0.54069 | 0.46356 | 0.56647 | 0.49116 | 0.59074 | 0.48193 | 0.57068 | 0.48252 | 0.88214 | 0.49580 |
| Sub#50 | 16 | 0.55673 | 0.48276 | 0.56809 | 0.53053 | 0.58011 | 0.47339 | 0.55121 | 0.50620 | 0.99062 | 0.47149 |
| Sub#51 | 16 | 0.51297 | 0.46448 | 0.51951 | 0.47755 | 0.54558 | 0.50714 | 0.54550 | 0.48946 | 0.86607 | 0.52088 |
| Sub#52 | 17 | 0.52091 | 0.46106 | 0.52978 | 0.47710 | 0.55193 | 0.49180 | 0.55697 | 0.49982 | 0.81532 | 0.45221 |
| Sub#53 | 17 | 0.57878 | 0.51040 | 0.59657 | 0.54232 | 0.62629 | 0.51571 | 0.58559 | 0.52184 | 1.07773 | 0.67126 |
| Sub#54 | 17 | 0.53114 | 0.45734 | 0.54801 | 0.50181 | 0.59604 | 0.52181 | 0.56663 | 0.51306 | 1.02462 | 0.52615 |
| Sub#55 | 18 | 0.48971 | 0.43571 | 0.51321 | 0.46894 | 0.50649 | 0.46048 | 0.53964 | 0.49240 | 0.83312 | 0.52325 |
| Sub#56 | 18 | 0.51544 | 0.46066 | 0.55325 | 0.51158 | 0.56295 | 0.47380 | 0.54038 | 0.46831 | 0.76838 | 0.48616 |
| Sub#57 | 19 | 0.50031 | 0.41366 | 0.53816 | 0.44108 | 0.54241 | 0.47460 | 0.53668 | 0.44794 | 0.80497 | 0.54952 |
| Sub#58 | 20 | 0.46820 | 0.42134 | 0.49179 | 0.44476 | 0.49538 | 0.41353 | 0.51646 | 0.44393 | 0.88948 | 0.48699 |
| Sub#59 | 21 | 0.52358 | 0.43879 | 0.53173 | 0.44354 | 0.52363 | 0.41787 | 0.53278 | 0.46061 | 0.85682 | 0.51996 |
| Sub#60 | 22 | 0.52783 | 0.47215 | 0.52498 | 0.48232 | 0.58533 | 0.50490 | 0.55993 | 0.50760 | 0.92723 | 0.59995 |
| Sub#61 | 22 | 0.49325 | 0.41837 | 0.50214 | 0.45182 | 0.58547 | 0.47638 | 0.54660 | 0.44323 | 0.77003 | 0.49712 |
| Sub#62 | 23 | 0.46430 | 0.41928 | 0.49830 | 0.44563 | 0.51270 | 0.42356 | 0.57708 | 0.48274 | 0.72199 | 0.46993 |
| Sub#63 | 23 | 0.50127 | 0.43178 | 0.52711 | 0.44693 | 0.48748 | 0.44438 | 0.50503 | 0.44030 | 0.94419 | 0.55494 |
| Sub#64 | 24 | 0.58836 | 0.49678 | 0.59863 | 0.54086 | 0.57820 | 0.47607 | 0.58688 | 0.48302 | 1.05110 | 0.71424 |
| Sub#65 | 25 | 0.54304 | 0.47539 | 0.54333 | 0.48231 | 0.59077 | 0.48317 | 0.67038 | 0.52784 | 0.98917 | 0.65709 |

Table 5: The absolute fiber length (AL) and normalized fiber length (NL) of each subject is shown. The values are shown for left (L) and right (R) cingulate gyrus part of cingulum (cgc), left and right cingulum hippocampal part (cgh) and fornix (fx). AL is measured in mm. This table shows the quantitative data of Fig. 7.

| **Sub ID** | **Age (Years)** | **cgc-L** | | **cgc-R** | | **cgh-L** | | **cgh-R** | | **fx** | |
| --- | --- | --- | --- | --- | --- | --- | --- | --- | --- | --- | --- |
|  |  | **AL** | **NL** | **AL** | **NL** | **AL** | **NL** | **AL** | **NL** | **AL** | **NL** |
| Sub#1 | 0.02 | 54.535 | 0.501 | 55.652 | 0.511 | 46.077 | 0.423 | 42.164 | 0.387 | 67.224 | 0.617 |
| Sub#2 | 0.03 | 41.808 | 0.428 | 45.934 | 0.470 | 58.000 | 0.593 | 53.000 | 0.542 | 59.486 | 0.608 |
| Sub#3 | 0.05 | 60.273 | 0.560 | 53.335 | 0.496 | 58.772 | 0.546 | 57.377 | 0.533 | 54.526 | 0.507 |
| Sub#4 | 0.42 | 83.479 | 0.684 | 74.782 | 0.613 | 81.635 | 0.669 | 70.882 | 0.581 | 75.762 | 0.621 |
| Sub#5 | 1.99 | 92.345 | 0.637 | 99.948 | 0.689 | 86.973 | 0.600 | 98.785 | 0.681 | 100.000 | 0.690 |
| Sub#6 | 2.17 | 107.741 | 0.691 | 103.193 | 0.661 | 91.698 | 0.588 | 85.271 | 0.547 | 104.000 | 0.667 |
| Sub#7 | 2.35 | 77.968 | 0.549 | 94.794 | 0.668 | 91.744 | 0.646 | 89.233 | 0.628 | 97.135 | 0.684 |
| Sub#8 | 2.50 | 116.762 | 0.840 | 129.849 | 0.934 | 85.416 | 0.615 | 87.132 | 0.627 | 89.000 | 0.640 |
| Sub#9 | 2.59 | 85.017 | 0.612 | 82.113 | 0.591 | 91.337 | 0.657 | 101.783 | 0.732 | 85.000 | 0.612 |
| Sub#10 | 3.21 | 84.107 | 0.550 | 89.278 | 0.584 | 85.278 | 0.557 | 78.906 | 0.516 | 86.000 | 0.562 |
| Sub#11 | 3.36 | 94.382 | 0.715 | 74.254 | 0.563 | 91.487 | 0.693 | 75.317 | 0.571 | 91.501 | 0.693 |
| Sub#12 | 3.58 | 101.526 | 0.725 | 87.058 | 0.622 | 102.069 | 0.729 | 102.709 | 0.734 | 93.164 | 0.665 |
| Sub#13 | 3.84 | 102.389 | 0.652 | 88.872 | 0.566 | 80.085 | 0.510 | 83.648 | 0.533 | 102.945 | 0.656 |
| Sub#14 | 4.02 | 104.398 | 0.687 | 80.779 | 0.531 | 80.085 | 0.510 | 94.241 | 0.620 | 77.877 | 0.512 |
| Sub#15 | 4.25 | 114.668 | 0.740 | 94.781 | 0.611 | 70.020 | 0.452 | 77.726 | 0.501 | 93.000 | 0.600 |
| Sub#16 | 4.39 | 115.700 | 0.761 | 93.126 | 0.613 | 103.053 | 0.678 | 104.913 | 0.690 | 86.084 | 0.566 |
| Sub#17 | 4.72 | 118.561 | 0.847 | 115.273 | 0.823 | 95.372 | 0.681 | 98.259 | 0.702 | 96.368 | 0.688 |
| Sub#18 | 5.02 | 126.131 | 0.841 | 99.331 | 0.662 | 77.624 | 0.517 | 73.819 | 0.492 | 99.474 | 0.663 |
| Sub#19 | 5.58 | 132.075 | 0.863 | 94.925 | 0.620 | 87.653 | 0.573 | 97.022 | 0.634 | 76.390 | 0.499 |
| Sub#20 | 5.75 | 107.098 | 0.724 | 105.127 | 0.710 | 59.727 | 0.404 | 91.484 | 0.618 | 114.000 | 0.770 |
| Sub#21 | 6.08 | 105.926 | 0.746 | 83.998 | 0.592 | 90.396 | 0.637 | 90.441 | 0.637 | 92.560 | 0.652 |
| Sub#22 | 6.42 | 126.016 | 0.869 | 91.945 | 0.634 | 81.598 | 0.563 | 80.487 | 0.555 | 79.979 | 0.552 |
| Sub#23 | 6.83 | 130.971 | 0.840 | 87.735 | 0.562 | 82.458 | 0.529 | 96.509 | 0.619 | 79.979 | 0.552 |
| Sub#24 | 7.00 | 112.879 | 0.743 | 92.542 | 0.609 | 91.534 | 0.602 | 85.641 | 0.563 | 85.639 | 0.563 |
| Sub#25 | 7.33 | 106.989 | 0.665 | 115.740 | 0.719 | 75.553 | 0.469 | 91.802 | 0.570 | 98.501 | 0.612 |
| Sub#26 | 7.50 | 127.121 | 0.831 | 123.802 | 0.809 | 94.776 | 0.619 | 97.488 | 0.637 | 101.000 | 0.660 |
| Sub#27 | 7.75 | 121.819 | 0.786 | 97.064 | 0.626 | 90.272 | 0.582 | 100.013 | 0.645 | 81.837 | 0.528 |
| Sub#28 | 8.08 | 135.188 | 0.845 | 103.807 | 0.649 | 72.917 | 0.456 | 100.160 | 0.626 | 86.000 | 0.538 |
| Sub#29 | 8.58 | 141.951 | 0.966 | 92.382 | 0.628 | 92.965 | 0.632 | 90.988 | 0.619 | 92.000 | 0.626 |
| Sub#30 | 8.75 | 102.166 | 0.695 | 110.496 | 0.752 | 93.240 | 0.634 | 80.820 | 0.550 | 91.800 | 0.624 |
| Sub#31 | 8.83 | 105.842 | 0.666 | 96.985 | 0.610 | 85.209 | 0.536 | 97.834 | 0.615 | 80.177 | 0.504 |
| Sub#32 | 9.17 | 101.600 | 0.643 | 92.183 | 0.583 | 94.610 | 0.599 | 98.854 | 0.626 | 101.927 | 0.645 |
| Sub#33 | 9.25 | 109.365 | 0.667 | 119.487 | 0.729 | 108.521 | 0.662 | 91.183 | 0.556 | 85.010 | 0.518 |
| Sub#34 | 9.50 | 105.559 | 0.704 | 102.432 | 0.683 | 88.321 | 0.589 | 86.807 | 0.579 | 84.988 | 0.567 |
| Sub#35 | 9.75 | 112.961 | 0.763 | 114.904 | 0.776 | 69.674 | 0.471 | 66.237 | 0.448 | 82.766 | 0.559 |
| Sub#36 | 10.08 | 89.050 | 0.641 | 95.172 | 0.685 | 94.003 | 0.676 | 100.768 | 0.725 | 80.024 | 0.576 |
| Sub#37 | 10.58 | 99.568 | 0.618 | 108.825 | 0.676 | 86.743 | 0.539 | 87.972 | 0.546 | 84.000 | 0.522 |
| Sub#38 | 10.83 | 120.739 | 0.805 | 94.023 | 0.627 | 84.701 | 0.565 | 109.740 | 0.732 | 89.522 | 0.597 |
| Sub#39 | 11.33 | 123.942 | 0.784 | 122.200 | 0.773 | 108.641 | 0.688 | 98.598 | 0.624 | 83.998 | 0.532 |
| Sub#40 | 11.75 | 122.968 | 0.848 | 101.267 | 0.698 | 98.961 | 0.682 | 94.534 | 0.652 | 87.453 | 0.603 |
| Sub#41 | 12 | 135.276 | 0.902 | 119.971 | 0.800 | 93.874 | 0.626 | 95.737 | 0.638 | 97.508 | 0.650 |
| Sub#42 | 12 | 104.616 | 0.657 | 126.763 | 0.796 | 84.654 | 0.532 | 84.845 | 0.533 | 81.162 | 0.510 |
| Sub#43 | 12 | 124.069 | 0.727 | 108.758 | 0.637 | 95.356 | 0.559 | 69.000 | 0.404 | 118.000 | 0.692 |
| Sub#44 | 12.25 | 129.364 | 0.840 | 98.540 | 0.640 | 107.545 | 0.698 | 102.503 | 0.666 | 126.000 | 0.818 |
| Sub#45 | 13 | 115.795 | 0.693 | 99.417 | 0.595 | 96.789 | 0.579 | 107.369 | 0.642 | 93.000 | 0.556 |
| Sub#46 | 14.08 | 115.062 | 0.715 | 97.875 | 0.608 | 107.239 | 0.666 | 88.566 | 0.550 | 87.542 | 0.544 |
| Sub#47 | 15 | 119.115 | 0.756 | 101.935 | 0.647 | 113.024 | 0.718 | 94.917 | 0.603 | 80.855 | 0.513 |
| Sub#48 | 15 | 121.927 | 0.753 | 110.347 | 0.682 | 84.750 | 0.524 | 75.007 | 0.463 | 111.000 | 0.686 |
| Sub#49 | 15.83 | 129.937 | 0.838 | 95.317 | 0.615 | 97.015 | 0.626 | 90.382 | 0.583 | 118.000 | 0.761 |
| Sub#50 | 16 | 117.253 | 0.698 | 121.861 | 0.725 | 102.603 | 0.611 | 91.156 | 0.543 | 82.793 | 0.493 |
| Sub#51 | 16 | 125.384 | 0.814 | 101.794 | 0.661 | 73.480 | 0.477 | 82.408 | 0.535 | 118.000 | 0.766 |
| Sub#52 | 17 | 131.985 | 0.762 | 113.718 | 0.656 | 107.025 | 0.618 | 102.639 | 0.592 | 124.000 | 0.716 |
| Sub#53 | 17 | 135.814 | 0.867 | 106.123 | 0.678 | 94.094 | 0.601 | 96.327 | 0.615 | 120.000 | 0.766 |
| Sub#54 | 17 | 121.014 | 0.717 | 123.291 | 0.730 | 92.354 | 0.547 | 93.751 | 0.555 | 114.000 | 0.675 |
| Sub#55 | 18 | 134.653 | 0.777 | 120.960 | 0.698 | 90.910 | 0.525 | 92.440 | 0.534 | 106.000 | 0.612 |
| Sub#56 | 18 | 118.061 | 0.692 | 117.755 | 0.690 | 91.541 | 0.537 | 85.878 | 0.503 | 119.000 | 0.697 |
| Sub#57 | 19 | 115.710 | 0.727 | 129.820 | 0.815 | 70.000 | 0.440 | 93.430 | 0.587 | 100.220 | 0.629 |
| Sub#58 | 20 | 108.910 | 0.662 | 113.040 | 0.687 | 90.120 | 0.548 | 75.790 | 0.461 | 116.360 | 0.707 |
| Sub#59 | 21 | 119.000 | 0.720 | 110.310 | 0.667 | 45.190 | 0.273 | 53.420 | 0.323 | 97.970 | 0.592 |
| Sub#60 | 22 | 156.488 | 0.836 | 129.391 | 0.691 | 122.155 | 0.652 | 101.958 | 0.544 | 98.059 | 0.524 |
| Sub#61 | 22 | 120.770 | 0.734 | 114.720 | 0.697 | 69.090 | 0.420 | 95.550 | 0.581 | 100.210 | 0.609 |
| Sub#62 | 23 | 122.680 | 0.783 | 100.500 | 0.642 | 66.240 | 0.423 | 106.160 | 0.678 | 70.410 | 0.450 |
| Sub#63 | 23 | 101.270 | 0.677 | 63.440 | 0.424 | 64.350 | 0.430 | 108.790 | 0.727 | 76.990 | 0.515 |
| Sub#64 | 24 | 132.242 | 0.813 | 115.394 | 0.709 | 105.892 | 0.651 | 98.026 | 0.602 | 123.000 | 0.756 |
| Sub#65 | 25 | 108.693 | 0.621 | 110.759 | 0.633 | 80.830 | 0.462 | 80.000 | 0.457 | 116.360 | 0.707 |
